# Relay-projection microscopic telescopy

**DOI:** 10.1038/s41377-025-01800-6

**Published:** 2025-03-07

**Authors:** Wenjun Yi, Shuyue Zhu, Meicheng Fu, Nan Gu, Junli Qi, Siyu Liu, Mengjun Zhu, Ping Wang, Xin Chen, Yi Zhang, Hongyu Zhang, Yao Xu, Junyi Du, Peng Xiong, Zhaohua Dong, Luobing Dong, Qiong Liu, Xiujian Li

**Affiliations:** 1 College of Science, National University of Defense Technology, Changsha, China; 2 Xi’an Satellite Control Center, Xi’an, China; 3 School of Information and Electronics Engineering, Hunan City University, Yiyang, China; 4 College of Electronic Science, National University of Defense Technology, Changsha, China; 5 The School of Computer Science and Technology, Xidian University, Xi’an, China; 6 Senior Department of Otolaryngology Head and Neck Surgery, the 6th Medical Center of Chinese PLA General Hospital, Chinese PLA Medical School, Beijing, China

**Keywords:** Imaging and sensing, Microscopy

## Abstract

The fundamental trade-off between spatial resolution and imaging distance poses a significant challenge for current imaging techniques, such as those used in modern biomedical diagnosis and remote sensing. Here, we introduce a new conceptual method for imaging dynamic amplitude-phase-mixed objects, termed relay-projection microscopic telescopy (rPMT), which fundamentally challenges conventional light collection techniques by employing non-line-of-sight light collection through square-law relay-projection mechanisms. We successfully resolved tiny features measuring 2.76 μm, 22.10 μm, and 35.08 μm for objects positioned at distances of 1019.0 mm, 26.4 m, and 96.0 m, respectively, from single-shot spatial power spectrum images captured on the relay screen; these results demonstrate that the resolution capabilities of rPMT significantly surpass the Abbe diffraction limit of the 25 mm-aperture camera lens at the respective distances, achieving resolution improvement factors of 7.9, 25.4, and 58.2. The rPMT exhibits long-distance, wide-range, high-resolution imaging capabilities that exceed the diffraction limit of the camera lens and the focusing range limit, even when the objects are obscured by a scattering medium. The rPMT enables telescopic imaging from centimeters to beyond hundreds of meters with micrometer-scale resolution using simple devices, including a laser diode, a portable camera, and a diffusely reflecting whiteboard. Unlike contemporary high-resolution imaging techniques, our method does not require labeling reagents, wavefront modulation, synthetic receive aperture, or ptychography scanning, which significantly reduce the complexity of the imaging system and enhance the application practicality. This method holds particular promise for in-vivo label-free dynamic biomedical microscopic imaging diagnosis and remote surveillance of small objects.

## Abstract (web summary)

This imaging method exceeds the diffraction limit of the camera lens used, achieving resolution improvement factors of 7.9, 25.4, and 58.2 for objects at distances of 1019.0 mm, 26.4 m, and 96.0 m.

## Graphical abstract

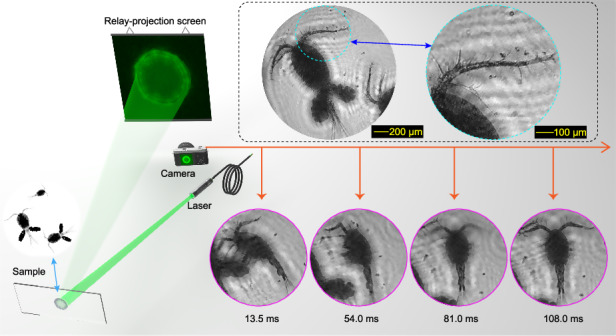

## Introduction

Modern biomedical diagnosis and remote sensing have reached a stage where wide-distance-range microscopic imaging in vivo and precise imaging monitoring of tiny objects at long distances are the unavoidable challenges faced by the scientific and industrial communities, which require high resolution, wide distance range and long distance to be simultaneously satisfied. However, the imaging capabilities of current imaging techniques, such as telescopy and microscopy, are limited by the fundamental trade-off between resolution and imaging distance, as well as imaging distance range, in finite aperture imaging systems. In other words, achieving high imaging resolution typically results in a short imaging distance and narrow distance range, whereas a long imaging distance is accompanied by low imaging resolution.

Numerous innovative imaging methodologies have focused on resolution improvement and capacity to image through scattering media^1–7^. These methodologies include lensless imaging methods like the coherent diffraction imaging (CDI)^8–10^ and the speckle correlation imaging^11,12^, synthetic receive aperture imaging methods such as the synthetic aperture lidar^13,14^ and the ptychographic imaging^15–19^, and stimulated fluorescence microscopic imaging methods like the stimulated emission depletion (STED)^1,20^, the Photo Activated Localization Microscopy (PALM)^21^, the Stochastic Optical Reconstruction Microscopy (STORM)^22^, and the DNA-Based Point Accumulation for Imaging in Nanoscale Topography (DNA-PAINT)^23^. However, the above imaging methods, along with the traditional imaging methods, are based on direct light collection configurations and are basically limited in their ability to fully address the trade-off between imaging resolution and imaging distance.

We demonstrate a relay-projection microscopic telescopy (rPMT) method based on indirect light collection configurations, which only requires a simple device kit comprised of a laser diode, a portable camera, and a white diffuse reflector to achieve high-resolution imaging. Our rPMT method challenges the conventional approach to light collection from objects. Instead of directly capturing light from the objects, our method involves capturing the enlarged relay-projection spatial power spectrum patterns, which allows for micrometer-scale resolution imaging of objects situated across a wide range of distances, spanning from microscopic to telescopic, using the same equipment. The imaging distances can vary from centimeters to more than hundreds of meters.

The rPMT method showcases long-distance, wide-range, high-resolution dynamic imaging capabilities that surpass the diffraction limit of the camera lens and the focusing range limit, through a single-shot capture and reconstruction, even when the objects are obscured by a scattering medium. Furthermore, distinguished from contemporary super-resolution techniques, our rPMT circumvents the necessity for labeling reagents, wavefront modulation^24–27^, synthetic receive aperture, and scanning in parallel^15–17,28,29^ or perpendicular^18,30,31^ to the propagation direction. These characteristics greatly reduce the complexity of the imaging system and enhances the practicality of the rPMT, broadening its potential application scope especially for dynamic imaging. In theory, rPMT can acquire almost arbitrarily large spatial power spectrum (SPS) images to achieve the desired spatial resolution. However, the actual SPS image captured by the readout camera is a sampled version of the continuous SPS intensity distribution projected onto the relay screen. During the discretized sampling process of the continuous SPS, the sampling interval, sampling number, and dynamic range are determined by the specifications of the readout camera employed, which significantly influence object reconstruction. Meanwhile, the signal-to-noise ratio (SNR) is influenced by a combination of factors, including the laser, camera, object, relay screen, and ambient light conditions. To facilitate successful object reconstruction from the sampled SPS image, a specific phase retrieval (PR) algorithm, termed the nonlinear-constraint Ping-Pong algorithm, is proposed for the optimal utilization of low-SNR high-frequency components and the tolerance enhancements for overexposure and low dynamic range of the SPS images, which also exhibits favorable interpolation properties and reduced dependence on sparse objects compared to other PR algorithms. Nonetheless, only the effective regions of the SPS image with sufficient SNR contribute to spatial resolution improvements of the retrieved object image.

The rPMT method demonstrates significant potential for long-distance, non-interference, non-contact microscale biomedical microscopic imaging for diagnosis and monitoring. Furthermore, it is well-suited for remote surveillance of small objects in extreme and radiation environments, where the camera needs to be positioned at a considerable distance from the objects and the imaging distance may vary dynamically over a wide range.

## Results

**Fig. 1:**
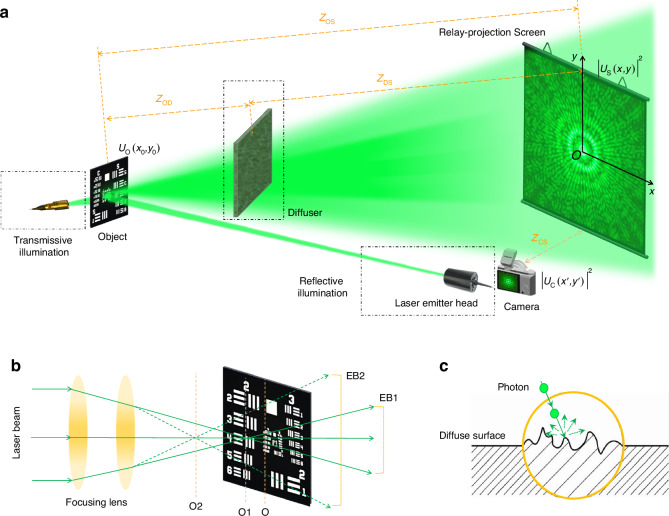
**The schematic diagram of rPMT**. **a** Overview of the method concept. **b** Illuminating laser beam profiles vary to accommodate different imaging requirements, with the illumination intensity and imaging area size on the object being adjustable through the focusing lens. EB1/EB2, expanded beams. **c** The diffuse surface of the relay screen scatters photons in all directions, indicating that the screen achieves recording and forwarding of the intensity of incident light in a statistical average perspective

As shown in Fig. 1a, the SPS pattern of an object is projected onto a diffusely reflecting screen of considerable size and then captured by a portable camera equipped with a wide-angle lens focusing on the screen. The imaging process can be carried out under either transmissive or reflective coherent illumination, denoted as the “Transmissive illumination” and “Reflective illumination” blocks, respectively. A diffuser will be placed between the object and the screen for scattering imaging experiments. In the following discussion, we will demonstrate that the object image can be reconstructed accurately with very high spatial resolution. The rPMT system’s spatial resolution limit is determined by the size of the relay-projection screen, significantly surpassing the diffraction limit of the camera lens aperture used. Depending on the objects and imaging requirements, the illumination mode (transmissive or reflective), projection distance (object-to-screen distance *Z*_OS_), and relay-projection screen size can be adjusted as needed. This flexibility allows the screen to be significantly larger than the camera lens in size.

The rPMT, inspired by the joint transform correlator^32,33^, incorporates an additional square-law transform to enhance the feasibility and practicality of resolution improvement. Unlike direct light collection configurations (refer to Supplementary Fig. S1a), where the camera must directly face and focus on the object to collect light, the rPMT utilizes indirect light collection with the additional square-law transform introduced by diffuse reflection of the relay-projection screen. This process involves projecting the object’s continuous SPS pattern onto a relay screen, followed by sampling the projected SPS using a readout camera. The surface diffuse reflection of the relay screen causes an equal probability of photon reflection in all directions (as illustrated in Fig. 1c). The SPS image captured by the readout camera represents a sampled/discretized version of the continuous SPS intensity distribution projected onto the relay screen, as it remains unaffected by phase effects during the secondary imaging process. Consequently, from an approximate analogy perspective, in addition to the square-law light transformation performed by the readout camera, the projection onto the screen can be regarded as an additional square-law transformation. In this discretized sampling process of the SPS, the sampling interval, sampling numbers, and dynamic range are determined by the specifications of the readout camera and the flexible distance between the camera and the relay screen.

The fundamental difference between the direct light collection configurations and the indirect configurations hinges on their respective methodologies. In a direct configuration, the process involves a single square-law light collection on the sensor based on coherent or incoherent superposition of line-of-sight light, either directly or facilitated by lenses and reflectors. Conversely, in an indirect configuration, the process can be conceptualized as a non-line-of-sight light collection process, which unfolds in two distinct steps: the first square-law relay-projection on the screen and the second square-law light collection on the sensor. This differentiation holds significant importance, as it profoundly influences the primary determinant governing imaging resolution capability and may bring about some special performance.

According to Abbe’s theory^34^, for the direct light collection configuration, the camera lens aperture (typically represented by the ratio of the aperture radius to the object distance) should be sufficiently large to gather light from objects with high spatial frequency components so as to produce high-resolution images. In other words, the imaging resolution is strictly determined by the lens aperture (detector array size for lensless imaging) and the object distance. While for the indirect-configuration rPMT method, the visible light from the object is first collected by a diffusing screen to create a light intensity pattern that can be visually observed by naked eyes. Subsequently, a camera with suitable wide-angle lens only needs to capture the light intensity pattern projected on the diffusing screen to provide data for the reconstruction algorithm. Therefore, the inherent limitations of the direct light collection configuration, which may be exceedingly challenging to overcome, may pose no issue for the indirect light collection configuration.

Herein, we propose a specific phase retrieval algorithm called the nonlinear-constraint Ping-Pong algorithm (NCPP) for the rPMT method, which can reconstruct the object images from the projected SPS images. Due to the degradations of signal-to-noise ratio (SNR) and dynamic range in the raw SPS image captured by indirect light collection configuration, the conventional PR algorithms such as the error reduction algorithm (ER)^35,36^, the hybrid input-output algorithm (HIO)^37,38^, and the Ping-Pong algorithm^39^ are not able to effectively reconstruct the accurate object image from a single-shot SPS pattern (refer to Supplementary Fig. S5). Herein, the NCPP algorithm integrates the Ping-Pong algorithm with a series of variable nonlinear modulus^40^ constraints. We have developed two versions of NCPP for different application scenarios: NCPP for Fraunhofer-diffraction (far-field approximation) SPS (refer to Algorithm 1 in Supplementary Section 3) and phase-modulated NCPP for Fresnel-diffraction (paraxial approximation) SPS (refer to Algorithm 2 in Supplementary Section 4).

Basically, the high spatial frequency components of the SPS pattern (located at large $(x,y)$ as shown in Fig. 1) correspond to the high-resolution features and hyperfine structures of the object for a certain object-to-screen distance. Meanwhile, a greater object-to-screen distance means larger size of SPS pattern on the screen and higher separation degree of the SPS structure features from an overlapping state. Owing to the inherent property of spatial nonlocality of light^41^, here the relay-projection screen functions as optical square-law converter and magnifier. This allows for the coupling of the object, screen, and camera over a long distance, thereby enhancing the flexibility of the imaging system to accommodate a wide range of applications, from microscopy to telescopy.

### rPMT-based microscopic imaging results

A negative USAF 1951 resolution target was positioned 1019.0 mm away from the relay screen to serve as a transmissive object, illuminated by an expanded laser beam (*λ* = *532* nm) under “Transmissive illumination” mode. An A0 size print paper (841 mm × 1189 mm) affixed to a white board served as the relay-projection screen, which could be considered as an approximate Lambert reflector. The projected SPS patterns on the screen were captured by a 4-megapixel monochrome camera with a fixed-focus imaging lens of 25 mm front aperture. Calibration of the observation-plane sampling interval on the relay screen can be achieved using a card with a known size, as detailed in the Materials and methods section.

**Fig. 2:**
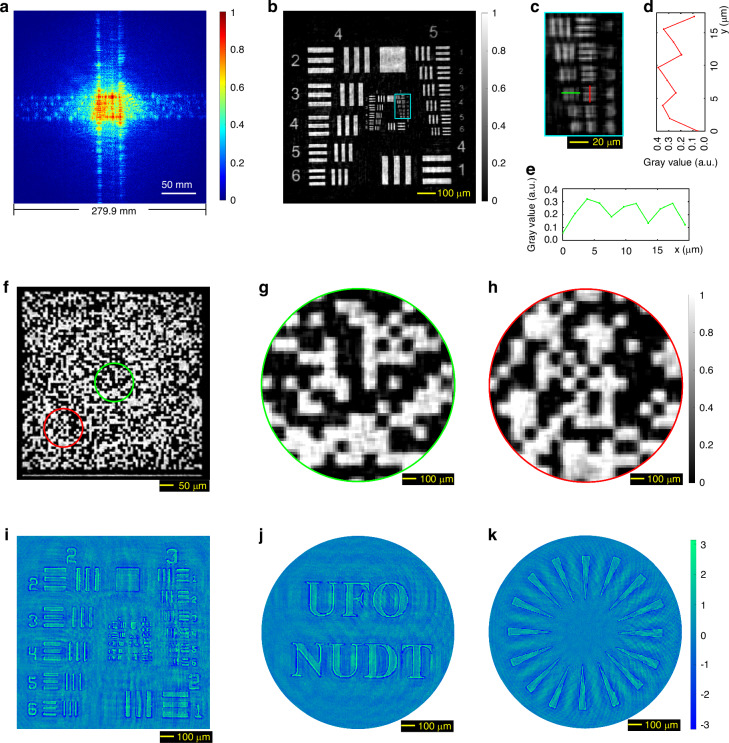
**Resolution capability test and phase contrast imaging of rPMT at a meter-scale object-to-screen distance**. **a** The normalized SPS image of a transmissive USAF 1951 resolution target at an object-to-screen distance of *Z*_OS_ = 1019.0 mm, with an exposure time of 150 ms. **b** Single-shot reconstruction from (**a**). **c** Detailed region (the elements in Group 7 of the USAF 1951 resolution target) cropped from (**b**). **d**, **e** The green and red line profiles correspond to the marks in (**c**) for the line pairs of Group 7 Element 4 with a line width of 2.76 μm. **f** The single-shot experimental result for a random-matrix encoding transmissive mask with an element size of 9.26 μm. **g**, **h** Two detailed regions cropped from (**f**). **i** The retrieved single-shot phase contrast image through rPMT using a phase object generated by a reflective-mode phase-only SLM. Here, the line widths of the resolution target image loaded into the phase-only SLM are not identical with those of a standard USAF 1951 target. Refer to Supplementary Fig. S6 for further details. **j**, **k** Another two retrieved phase contrast images

As shown in Fig. 2a single-shot SPS image (Fig. 2a), with an actual side length of ${L}_{{\rm{s}}}=279{\rm{.9mm}}$ on the relay screen, provides sufficient spatial frequency data for the phase-modulated NCPP algorithm (Algorithm 2 in Supplementary Section 4) to achieve a fine reconstruction, as illustrated in Fig. 2b, whose detailed region is depicted in Fig. 2c. As depicted in Fig. 2d, e, the reconstruction successfully resolves the line pairs of Group 7 Element 4 (referred to as G7E4) with a line width of 2.76 μm (refer to Supplementary Table S1 for further details), which is far beyond the Abbe diffraction-limited line width (only about $21{\rm{.68}}{\rm{\mu }}{\rm{m}}$) of the 25 mm-aperture camera lens used, i.e., achieving a resolution improvement factor of 7.9; it should be noticed that the achieved resolvable feature is smaller than half of the 6.5 μm pixel size of the imaging sensor used. In fact, the rPMT achieve a resolvable line width close to the theoretical diffraction-limited line width of the rPMT system determined by the size of the relay screen, i.e., ${\delta }_{{\rm{s}}}=\lambda {Z}_{{\rm{OS}}}/{L}_{{\rm{s}}}=1{\rm{.94}}{\rm{\mu }}{\rm{m}}$. The practical resolvable line width is larger than the theoretical value, due to the limited SNR and dynamic range of the SPS image.

Figure 2f displays the single-shot rPMT result of a random-matrix encoding transmissive mask with $70\times 70$ matrix elements and each element size of 9.26 μm×9.26 μm, which is considered a complex structural amplitude target. It can be observed that each element of the mask matrix is accurately retrieved (refer to Supplementary Fig. S10 for ground truth), which demonstrates that our rPMT method is capable of achieving high-resolution imaging not only for sparse targets but also for complex targets.

To evaluate the rPMT’s capability in imaging phase objects, we conducted a series of experiments using phase objects generated by a reflective-mode phase-only Spatial Light Modulator (SLM, HOLOEYE PLUTO, 1920 × 1080 pixels, 8 µm pixel pitch size, 8-bit gray levels). As depicted in Supplementary Fig. S6, various images (Supplementary Fig. S6c, g, i) were loaded onto the SLM to serve as phase objects, which were illuminated by the 532 nm laser diode. Subsequently, the SPS patterns were projected onto a relay screen and captured by the same monochrome camera and imaging lens above. As shown in Fig. 2i, the phase object is faithfully reconstructed with high resolution using the NCPP algorithm. The additional results, presented in Fig. 2j, k, further support this conclusion.

**Fig. 3:**
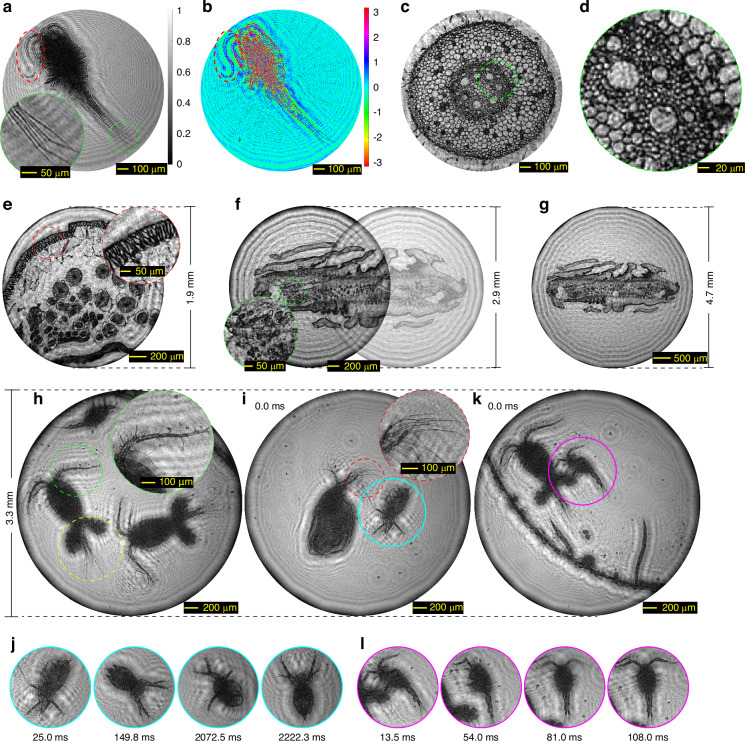
**rPMT-based single-shot microscopic imaging for biological microscope slides and dynamic live samples at a meter-scale working distance**. **a** The retrieved amplitude (i.e., the normalized transmittance) of a cyclop at an object-to-screen distance of *Z*_OS_ = 913.0 mm. The detailed region marked by the green dotted circle reveals clear identification of each caudal rami of the cyclop. **b** The retrieved phase contrast image of the cyclop, providing insight into high-transmittance regions in (**a**), such as the region indicated by the red dotted ellipse. The phase information can aid in the identification of these regions. **c** Transverse section of a young root tip of broad bean at *Z*_OS_ = 1084.3 mm. **d** Detailed region cropped from (**c**). **e** The transverse section of a female roundworm with a FOV of 1.9 mm diameter. The detailed region marked by the red dotted circle clearly identifies the longitudinal muscle cells. **f** Two sections of a single tenia proglottid measuring 3.9 mm in length, with a FOV of 2.9 mm diameter. The detailed region marked by the green dotted circle clearly identifies the vacuoles of the cytoplasmic region. **g** The same tenia proglottid illustrated in (**f**), situated within a single FOV of 4.7 mm diameter at *Z*_OS_ = 1072.2 mm. **h** The retrieved image of three live cyclops with a 3.3 mm-diameter FOV at *Z*_OS_ = 1080.5 mm. The region indicated by the green dotted circle distinctly highlights the antennal spines on the first antenna, whereas the yellow dotted circle reveals clear caudal ramus and the egg sacs. **i** One frame from a reconstructed video featuring a live Moina mongolica (indicated by the blue solid circle) alongside a Simocephalus vetulus, which SPS image sequence was captured at a frame rate of 40 fps (refer to Supplementary Video 1 for the full reconstructed video). The region indicated by the red dotted circle distinctly highlights the rod-shaped antennal spines on the antenna of the Simocephalus vetulus. **j** Four additional frame of the video, captured at 25.0 ms, 149.8 ms, 2072.5 ms, and 2222.3 ms, subsequent to the frame illustrated (**i**). These figures exclusively display the ROIs, indicated by the blue solid circles in **i**, which illustrate the postural changes of Moina mongolica. **k** One frame from a reconstructed video showcasing two live cyclops alongside two Caenorhabditis elegans, which SPS image sequence was captured at 74 fps (refer to Supplementary Video 1 for the full reconstructed video). **l** Four additional frame of the video, captured at 13.5 ms, 54.0 ms, 81.0 ms, 108.0 ms following the frame shown in (**k**). These figures focus exclusively on the ROIs, denoted by the pink solid circles in (**k**), which clearly illustrate the jumping process of the small cyclop on the egg sacs of the larger cyclop with a time sampling interval of 13.5 ms. (refer to Supplementary Video 1 for details)

To assess the practicality and resolution capabilities of rPMT in microscale biomedical imaging, we conducted rPMT-based microscopic imaging on a series of amplitude-phase-mixed biological microscope slides (Fig. 3a–g) and dynamic live samples (Fig. 3h–l) positioned at a meter-scale object-to-screen distance. The single-shot experimental results are presented in Fig. 3.

Specifically, Fig. 3a, b display the retrieved amplitude (i.e., normalized transmittance) and phase contrast image of a complete cyclop specimen at an object-to-screen distance of *Z*_OS_ = 913.0 mm. The detailed region of interest (ROI) marked by the green dotted circle in Fig. 3a is magnified, revealing clear identification of each caudal rami of the cyclop. The phase contrast image in Fig. 3b aids in identifying high-transmittance regions in Fig. 3a, such as the area marked by the red dotted ellipse, which corresponds to the antenna of the cyclop. Figure 3c displays the transverse section of a young root tip of broad bean at *Z*_OS_ = 1084.3 mm, whose detailed region is shown in Fig. 3d. The results demonstrate a remarkable capacity of rPMT for resolving intricate biological structural details alongside the ability to discern subtle grayscale and phase variations across distinct fine regions, thereby indicating high-resolution imaging capabilities for irregular biological specimens.

Figure 3e depicts the transverse section of a female roundworm with a FOV of 1.9 mm diameter. The detailed region marked by the red dotted circle clearly identifies the longitudinal muscle cells of this roundworm. Figure 3f shows two sections of a single tenia proglottid with a length of 3.9 mm, with a FOV of 2.9 mm diameter. The detailed region marked by the green dotted circle clearly identifies the vacuoles of the cytoplasmic region. Furthermore, to achieve a FOV that encompasses the entire tenia proglottid, one strategy is to enlarge the diameter of the illumination diaphragm. However, this may result in reconstruction failures if the FOV diameter exceeds the overall size of the object domain. To address this challenge, we propose applying $\alpha \times$ interpolation upsampling (where $\alpha > 1$ denotes the upsampling factor) to the SPS image prior to its reconstruction with the NCPP algorithm. This method can effectively enlarge the object domain size by a factor of $\alpha$, thereby ensuring it covers the FOV. For more details, please refer to the “Image Pre-processing” section in the Materials and Methods. Figure 3g shows the same tenia proglottid illustrated in Fig. 3f, but situated within a single FOV of 4.7 mm diameter at *Z*_OS_ = 1072.2 mm. These results demonstrate the ability of rPMT to achieve high resolution and large FOV microscopic imaging simultaneously.

In the experiments involving live samples, the specimens are placed in micro culture dishes with a depth of 0.5 mm and a diameter of 6 mm, which are put *Z*_OS_ = 1080.5 mm away from the relay screen. Figure 3h illustrates the retrieved image of three live cyclops with a 3.3 mm-diameter FOV. The region marked by the green dotted circle distinctly highlights the antennal spines on the first antenna, whereas the yellow dotted circle reveals clear caudal ramus and the egg sacs.

Figure 3i presents one frame from a reconstructed video featuring a live Moina mongolica (indicated by the blue solid circle) alongside a Simocephalus vetulus. The SPS image sequence was captured at a frame rate of 40 frames per second (fps), and the full reconstructed video can be found in Supplementary Video 1. The region indicated by the red dotted circle distinctly highlights the rod-shaped antennal spines on the antennas of the Simocephalus vetulus. Figure 3j presents an additional four frames from this video, captured at 25.0 ms, 149.8 ms, 2072.5 ms, and 2222.3 ms following the frame shown in Fig. 3i. These figures exclusively display the ROIs, indicated by the blue solid circles in Fig. 3i, which illustrate the postural changes of the Moina mongolica. (refer to Supplementary Video 1 for details). Figure 3k illustrates one frame from a reconstructed video showcasing two live cyclops alongside two Caenorhabditis elegans. To enhance the frame rate and capture more dynamic features, the SPS image sequence was acquired in 2 × 2 binning mode at 74 fps, which represents the maximum frame rate achievable by the camera under these conditions. The full reconstructed video can be found in Supplementary Video 1. Figure 3l presents an additional four frames from the video, captured at 13.5 ms, 54.0 ms, 81.0 ms, 108.0 ms after the frame depicted in Fig. 3k. These figures focus exclusively on the ROIs, denoted by the pink solid circles in Fig. 3k, which clearly illustrate the jumping behavior of the small cyclop on the egg sacs of the larger cyclop with a time sampling interval of 13.5 ms (refer to Supplementary Video 1 for details).

Generally, the temporal dynamic features of retrieved live sample images are primarily influenced by the acquisition frame rate, which is limited by the capabilities of the camera hardware. Additionally, video reconstruction is a time-consuming process. The Python implementations of the NCPP algorithm were executed on a GPU-equipped notebook computer (the detailed configuration can be found in Section 11 of the Supplementary materials). By averaging the reconstruction times of 200 frames, we determined that the average reconstruction time per frame can be reduced to 0.827 seconds through parallel GPU computing. The runtimes under various conditions are presented in Supplementary Table S2. Consequently, when integrated with higher performance parallel computing device in the future, the rPMT will be able to achieve real-time imaging.

Different from conventional microscopy relying on a large numerical aperture and short working distance lens, our rPMT enables meter-scale working distance microscopy, which allows us to achieve high spatial resolution beyond the diffraction limit of the camera lens aperture while maintaining a large FOV. Notably, our method stands apart from current super-resolution imaging techniques as it does not require synthetic aperture, wavefront modulation, or ptychography scanning. In conclusion, through single-shot measurement and reconstruction, the rPMT achieves high spatial resolution and expansive FOV microscopic imaging with a meter-scale working distance. This capability is particularly advantageous for dynamic live samples, enabling the rPMT to produce image sequences with both high spatial and temporal resolution through the acquisition of SPS sequences with a high frame rate camera.

### rPMT-based telescopic imaging results

Using the same monochrome camera and imaging lens above, a series of reflective and transmissive objects, illuminated by an expanded laser beam from a 532 nm laser diode, were positioned at distances of 26.4 m and 96.0 m from a white wall serving as a relay-projection screen. The rPMT-based telescopic imaging experiments were conducted in “Reflective illumination” and “Transmissive illumination” modes.

**Fig. 4:**
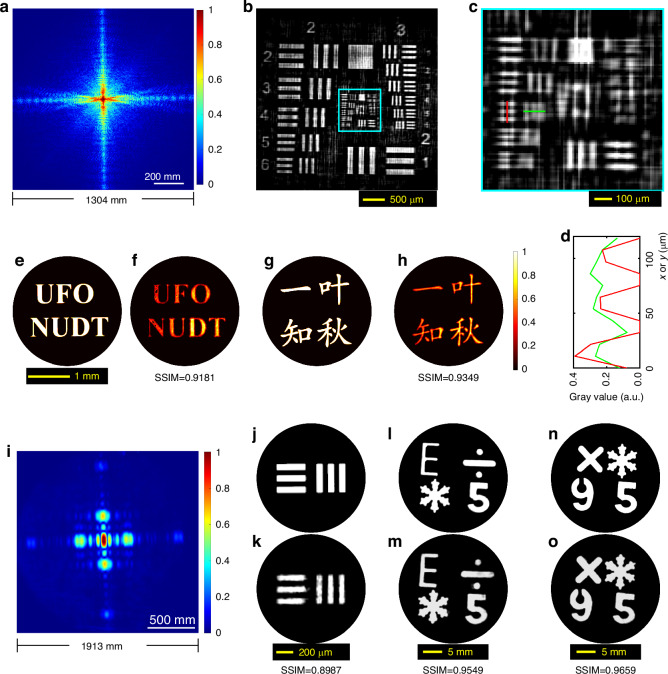
**Experiments of rPMT at object-to-screen distances of** ***Z***_**OS**_ = **26.4** **m and** ***Z***_**OS**_ = **96.0** **m**. **a** The normalized SPS image of a reflective USAF 1951 resolution target at *Z*_OS_ = 26.4 m (fusing two SPS images with exposure times of 700 μs and 40 ms). **b** Reconstruction from (**a**). **c** Detailed region (Groups 4,5 of the USAF 1951 resolution target) cropped from (**b**). **d** The red and green line profiles correspond to the marks in (**c**) for the line pair of Group 4 Element 4 with a line width of 22.10 µm. **e**, **g** Two original transmissive objects used. **f**, **h** The corresponding reconstructions from the SPS patterns captured at *Z*_OS_ = 26.4 m. **i**, The SPS image at *Z*_OS_ = 96.0 m of the transmissive object in (**j**). **j** The line pairs in Group 2 Element 6 of a transmissive USAF 1951 resolution target with a line width of 70.15 μm. **k** Object image retrieved from (**i**). **l**, **n** Two handmade reflective objects. **m** and (**o**), The corresponding reconstructions from the SPS patterns captured at *Z*_OS_ = 96.0 m

The first set of experiments were conducted at an object-to-screen distance of 26.4 m. Firstly, a positive USAF 1951 resolution target acted as the reflective target, and a dual-exposure fusion^42,43^ was performed to improve the dynamic range of the SPS pattern, by fusing two SPS images with exposure times of 700 μs and 40 ms to yield one composite SPS image with higher dynamic range (Fig. 4a). The reconstructed result and the detailed region (Groups 4 and 5) are respectively shown in Fig. 4b, c. Referring to the line widths of the USAF 1951 target in Supplementary Table S1, the best resolvable feature is G4E4 (Fig. 4c, d) with a line width of 22.10 µm, far beyond the Abbe diffraction-limited line width (about 561.79 μm) of the 25 mm camera lens aperture at 26.4 m, i.e., achieving a resolution improvement factor of 25.0. Here, the practical size of Fig. 4a on the relay-projection screen is ${L}_{{\rm{s}}}=1304{\rm{.3mm}}$, which yields a diffraction-limited line width of $10{\rm{.77}}{\rm{\mu }}{\rm{m}}$ in theory. Besides, two transmissive objects (Fig. 4e, g) served for the test, and the corresponding results are shown in Fig. 4f, h. The structural similarities (SSIM, 100% represents two identical images) between the retrieved object images and the original object images are both larger than 91%, which demonstrate that even the fine structure features such as the font features of the English letters and the Chinese idiom are faithfully retrieved. The rPMT method demonstrates a high-resolution telescopic imaging capability at the micrometer scale, surpassing that of the camera lens aperture. This is due to the adequate spatial separation and scale magnification of the projected SPS pattern, which can prevent mutual interference between adjacent light spots during acquisition^9^.

**Fig. 5:**
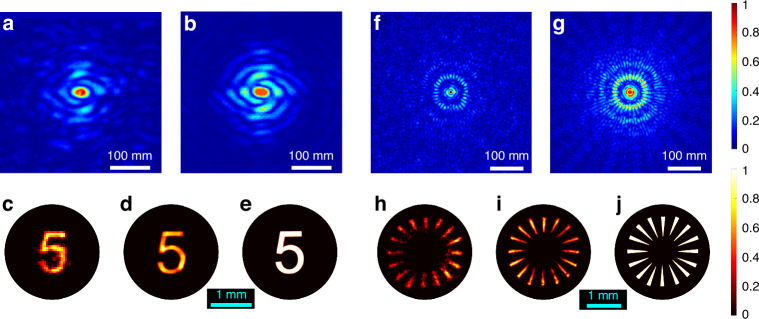
**Single-shot scattering imaging by rPMT at a diffuser-screen distance of** ${Z}_{{\rm{DS}}}=26{\rm{.0m}}$. **a** The normalized scattered SPS image of the transmissive object ‘5’. **b** The normalized scattering-free SPS image of the object ‘5’. **c**, **d** Sing-shot reconstructions from **a** and (**b**), respectively. **e** The original object ‘5’. **f**, **g** The normalized scattered and scattering-free SPS images of the second transmissive object. **h** and (**i**), Sing-shot reconstructions from **f** and (**g**), respectively. **j** The second transmissive object: an inhomogeneous star sample

The second set of experiments were performed to illustrate the resolution capability at an object-to-screen distance of *Z*_OS_ = 96.0 m, which is a telescopic distance for imaging micrometer-scale objects. A group of line pairs with a line width of 70.15 μm (Group 2 Element 6 of a negative USAF 1951 resolution target, as shown in Fig. 4j) served as the transmissive object, and the corresponding SPS image and reconstruction are shown in Fig. 4i, k, respectively. The clear separation of these line pairs indicates the resolution is better than 35.08 μm, far beyond the diffraction-limited line width (about 2.04 mm) of the 25 mm camera lens aperture, achieving a resolution improvement factor of 58.2. In practice, the resolution capability is close to the theoretical value corresponding to the relay screen size ${L}_{{\rm{s}}}=1912{\rm{.7mm}}$ (the actual size of Fig. 4i), i.e., ${\delta }_{{\rm{s}}}=26{\rm{.70}}{\rm{\mu }}{\rm{m}}$. Next, several centimeter-scale handmade reflective objects (Fig. 5l, n, refer to Supplementary Fig. S11 for details) were placed at an object-to-screen distance of *Z*_OS_ = 96.0 m for the rPMT experiments. The retrieved images are respectively shown in Fig. 5m, o. Both the SSIM indices are greater than 0.95, indicating high-fidelity reconstructions. We also use a smartphone to perform the measurements, and the results are shown in Fig. S9 of Supplementary Section 7.

### rPMT for imaging through scattering media

Biomedical diagnosis and remote sensing often require imaging through scattering media, such as the surface layers of biological tissue and cloud-mist layers. The following experiment results will demonstrate that the rPMT can help to achieve high-resolution imaging through scattering media across an ultra-wide range. Basically, the integration of the optical memory effect^44–46^ and Fourier optics guarantees that the scattered SPS of an object is essentially identical to the scatter-free counterpart^47^, especially in the low and mid-frequency components (refer to Supplementary Section 6 for further details).

As shown in Fig. 1a, a ground-glass diffuser (Thorlabs DG20-600-MD) working as a scattering medium layer was inserted into the light path at a position ${Z}_{{\rm{OD}}}=4{\rm{.0cm}}$ from the object (Fig. 5e) and ${Z}_{{\rm{DS}}}=26{\rm{.0m}}$ from the relay screen. The captured SPS patterns under two conditions, i.e., with and without the diffuser, are shown in Fig. 5a, b, respectively. These figures indicate the consistency between the scattered SPS and the scattering-free SPS, especially for the low and middle frequency components. Despite the low SNR and the submersion of high-spatial-frequency components by speckle noises in the scattered SPS pattern (Fig. 5a), the object image can still be clearly retrieved from a single-shot scattered SPS pattern by the NCPP algorithm (Algorithm 1 in Supplementary Section 3), as demonstrated in Fig. 5c. Another set of results is presented in Fig. 5f–j, supporting the same conclusion. To the best of our knowledge, our rPMT has the capability to extend the scattering imaging distance by one to two orders of magnitude, in contrast to the existing scattering imaging techniques, which typically works within a range of 1 meter or less.

## Discussion

The above results demonstrate that the rPMT can effectively achieves super-resolution imaging of microscale amplitude-phase-mixed objects across a wide range of object-to-screen distances spanning from centimeters to more than hundreds of meters, to provide snapshot images and dynamic videos. This capability effectively merges the functionalities of a microscope and a telescope using the same device. It should be noted that the results presented above have not yet reached the resolution limit of the rPMT, primarily due to technological limitations of current equipment. High-SNR and high-dynamic-range raw SPS patterns containing high-frequency components are crucial for retrieving high-fidelity super-resolution object images. This can be enhanced by employing a high-power pulsed laser and a range-gated camera equipped with an appropriate FOV lens and a high-sensitivity, high-dynamic-range, low-dark-current-noise, and large-scale image sensor. In addition, a large-sized relay screen with uniform and high diffuse reflectance is essential for long-distance super-resolution imaging. Last but not the least, the imaging sensor pixel size should be small to prevent the overlapping of high-spatial-frequency components, which is necessary for distinguishing extremely close and tiny components in the SPS patterns during the retrieval process. Here the successful rPMT through scattering media should be attributed to the robust statistical square-law transformation and the significant scale magnification by the relay-screen, similar to the strength of the optical joint transform correlator that is much more robust than the optical matched filter based on the 4-f optical structure.

In addition to the white print paper and indoor white painted wall we used, a variety of diffusely reflecting flat surfaces can serve as relay-projection screens, including projector screens, common off-the-shelf lime walls of buildings, water curtains, and even cloud layers in the sky. This versatility makes the rPMT a practical, robust, and cost-effective dynamic imaging solution for challenging and hard-to-reach imaging scenarios. The large FOV required for large-scale SPS acquisitions of the rPMT is more easily achieved than the large-size lens aperture, allowing a smartphone with a small entrance aperture but large FOV to support the rPMT.

Unlike contemporary super-resolution techniques, our rPMT method does not necessitate wavefront modulation, synthetic receive aperture, or ptychographic scanning. The dependence on these approaches can compromise the system’s ability to image dynamic objects and reduce its capacity for real-time processing. By utilizing single-shot measurement and reconstruction, rPMT achieves high spatial resolution and a wide FOV for microscopic imaging at extended distances. This capability is especially beneficial for dynamic live samples, allowing rPMT to generate image sequences with both high spatial and temporal resolution by acquiring SPS sequences at a frame rate determined by the detector. Furthermore, rPMT has the potential to be integrated with other computational imaging techniques. For example, it may be combined with ptychographic scanning or angular scanning of coherent illumination across various subregions of the objects to achieve an even larger FOV. Additionally, rPMT can be integrated with compressed sensing imaging to enhance the resolution of the captured SPS images, taking full advantage of the inherent sparsity of SPS images.

In summary, owing to the scale enlargement of SPS patterns and the additional square-law transform introduced by the relay projection, the rPMT can significantly improves spatial resolution, imaging distance, and distance range simultaneously. Therefore, the rPMT method is in line with the development trend of modern imaging technology, which emphasize higher spatial and temporal resolution, broader imaging distance ranges, unrestricted imaging distances in challenging scenarios, as well as the miniaturization and intelligence of imaging equipment. The advancement of the rPMT will provide new solutions for addressing challenges in various dynamic imaging applications, such as surgical microscopes for ENT, label-free biomedical microscopic imaging diagnosis, and precise imaging monitoring of small objects at long distances. These applications require ultra-wide working distance microscopy, long-range tiny target detection, and long-distance scattering imaging to be integrated into a single imaging task. In the future, the advancements in imaging sensors characterized by higher sensitivity, smaller pixel pitch, larger pixel array, lower dark current, and greater dynamic range are expected to enhance the performance and practicality of the rPMT. These improvements will enable the capture of more high-spatial-frequency components of the SPS patterns, ultimately enhancing the quality of the object images retrieved.

## Materials and methods

### Imaging instrumentation

In this study, the laser used for coherent illumination was a 2 W 532 nm continuous-wave laser diode (YN-532nm-2W-CC30478 LD pumped all-solid-state green laser, Yanban laser). A monochrome camera (Dhyana 400BSI, 2048 × 2048 pixels, 6.5 μm pixel size, 88 dB dynamic range, 16 bit depth) with an imaging lens (Thorlabs MVL50TM23, F/2.8, fixed focal length 50 mm, front aperture 25 mm) was used for all the SPS image acquisitions in the main text. The transmissive objects for imaging included a negative USAF 1951 resolution target (GCG-020602, Daheng optics) and several negative objects on a polyester photomask, while the reflective objects included a positive USAF 1951 target (GCG-020601, Daheng optics) and groups of holes of various shapes made on mirrors. In Figs. 2 and 3, the objects were illuminated using coherent spherical waves generated by defocused illumination of an achromatic lens (GCL-010616, Daheng optics) with a focal length of 150 mm; in Figs. 4 and 5, the objects were illuminated using coherent planar waves. The diffuser for scattering imaging was a 600-grit ground-glass diffuser (DG20-600-MD, Thorlabs).

The long-range experiments were performed in a 97-meter-long corridor, and the white lime walls at its both ends served as the relay-projection screens; the objects were placed at distances of 26.4 m or 96.0 m from the screen. For the ultra-wide working distance microscopic imaging (Figs. 2 and 3), a A0 print paper (841 mm × 1189 mm) pasted on a white board worked as the relay-projection screen. The diffracted light of the transmissive or reflective objects was projected onto the relay-projection screen to form SPS patterns, which were captured by the camera focused on the screen. The camera integration time varied in different experiments, typically a few hundred milliseconds for Figs. 3–4 and 8 s for Fig. 5a–f.

In order to achieve an appropriate FOV and sampling interval for the SPS acquisitions, the camera-to-screen distances ${Z}_{{\rm{CS}}}$ were adjusted in different scenarios. The observation plane, i.e., the relay-projection screen plane, and the object plane are related by $\Delta {x}_{0}=\lambda {Z}_{{\rm{OS}}}/(N\Delta x)$ (refer to Supplementary Eq. S3), in which $\Delta {x}_{0}$ and $\Delta x$ are respectively the sampling intervals of the object plane and the observation plane, *λ* is the wavelength of illuminating light, *Z*_OS_ is the object-to-screen distance as shown in Fig. 1a, and *N* is the row or column sampling number. According to the relationship above, for a fixed *N*, an undersized $\Delta x$ will lead to a larger object-plane interval $\Delta {x}_{0}$ as well as a smaller spatial frequency bandwidth, which results in a worse resolution limit; on the other hand, an oversized $\Delta x$ will lead to a smaller object-plane side length $N\Delta {x}_{0}$ and consequently increases the object’s area percentage within the object plane (as well as leads to an undersized Fresnel number^48,49^ in the SPS pattern), which will cause the single-shot reconstruction degradation and even failure (when the total object-plane size $N\Delta {x}_{0}$ is smaller than the object size).

Empirically, in order to achieve a high-quality single-shot reconstruction, the object’s side length, i.e., the side length of the effective FOV (object size), requires to be less than a proportion (*η*)of the entire object-plane side length $N\Delta {x}_{0}$. In detail, to achieve an expected resolution limit better than $\delta$, $\Delta x$ should meet $\Delta x\ge 2\lambda {Z}_{{\rm{OS}}}/(N\delta )$; to ensure the object’s approximate size ${l}_{{\rm{obj}}}$ is less than *η* ($0\, <\, \eta \le 1$) of the entire object-plane side length, $\Delta x$ should meet $\Delta x\le \lambda {Z}_{{\rm{OS}}}\eta /{l}_{{\rm{obj}}}$; in sum, a proper camera-to-screen distance ${Z}_{{\rm{CS}}}$ should satisfy $2\lambda {Z}_{{\rm{OS}}}f/(N\delta \Delta {x}_{{\rm{pix}}})\le {Z}_{{\rm{CS}}}\le \lambda {Z}_{{\rm{OS}}}\eta f/({l}_{{\rm{obj}}}\Delta {x}_{{\rm{pix}}})$ by taking into account $\Delta x\approx {Z}_{{\rm{CS}}}\Delta {x}_{{\rm{pix}}}/f$, in which $\Delta {x}_{{\rm{pix}}}$ is the sensor’s pixel size and *f* is the focus length of the camera lens. As described above, the camera-to-screen distance ${Z}_{{\rm{CS}}}$ has a high robustness. According to the camera-to-screen distance estimation method above, with respect to the monochrome camera and lens used, the practical ${Z}_{{\rm{CS}}}$ were respectively set to 1008 mm for Fig. 2a, 920 mm for Fig. 2f and Fig. 3, 4.8 m for Fig. 4a, 5.3 m for Fig. 4f, h, 9.7 m for Fig. 4i, and 2.0 m for Fig. 4m, o and Fig. 5.

### Object-plane sampling interval calibration

For the reconstructions of the SPS patterns in Figs. 2 and 3, and Fig. 4b, m, o, the spherical phase term in the Fresnel diffraction integral, i.e., $\Psi ({x}_{0},{y}_{0})=\exp [j\pi ({{x}_{0}}^{2}+{{y}_{0}}^{2})/(\lambda {Z}_{{\rm{OS}}})]$ (Supplementary Eq. S1), needs to be pre-estimated, in which the object-to-screen distance *Z*_OS_ can directly be premeasured using a laser rangefinder or estimated roughly due to the strong robustness of *Z*_OS_ in the reconstruction process. The object-plane sampling interval $\Delta {x}_{0}$ requires to be calibrated. For this purpose, we used a reference object with a known size, such as an identity card or a standard A4 paper, which was attached to the center of the relay-projection screen, to calibrate the observation-plane sampling interval $\Delta x$. The observation-plane sampling interval $\Delta x$ can be derived from $\Delta x={L}_{{\rm{ref}}}/{N}_{{\rm{ref}}}$, in which ${L}_{{\rm{ref}}}$ is the actual side length of the reference object and ${N}_{{\rm{ref}}}$ is its corresponding pixel number, and then $\Delta {x}_{0}$ can be obtained by $\Delta {x}_{0}=\lambda {Z}_{{\rm{OS}}}/(N\Delta x)$. It should be pointed out that, for the far-field SPS reconstructions in Fig. 4e–k and Fig. 5, neither calibration nor pre-measurement was required.

### Image pre-processing

The SPS pattern was cropped to an effective rectangular window with a dimension ranging from 1000 × 1000 pixels to 2048 × 2048 pixels, ensuring that the high-spatial-frequency gray values within the window needed to exceed a specific threshold, such as double the background value. A common way for background subtraction involves acquiring a separate background image without the SPS patterns; however, here we simply averaged the gray values over hundreds of pixels at a corner of the raw SPS image captured to generate a background value, and then subtracted it from the SPS window. The square root of the background-subtracted windowed SPS patterns, i.e., the modulus of the Fourier spectrum, served as the inputs of the subsequent NCPP phase retrieval algorithm. In the experiments with a smartphone, the raw RGB images were first converted to grayscale images. For the reconstruction in Fig. 4b, a dual-exposure fusion process was first performed simply by replacing the overexposed pixels’ gray values in the long-exposure SPS image with ${\tau }_{{\rm{long}}}/{\tau }_{{\rm{short}}}$ times of the corresponding pixels’ gray values in the short-exposure SPS image, so as to yield a fused SPS image with a higher dynamic range (Fig. 4a), where ${\tau }_{{\rm{long}}}$ and ${\tau }_{{\rm{short}}}$ were respectively the exposure times of the long-exposure and short-exposure SPS images. All the other reconstructions were performed on single-shot SPS images.

When the FOV diameter ${D}_{{\rm{FOV}}}$ exceeds the overall size of the object domain $N\Delta {x}_{0}$, reconstruction failures may occur. To mitigate this issue, we propose employing $\alpha \times$ interpolation upsampling (where $\alpha \ge {D}_{{\rm{FOV}}}/N\Delta {x}_{0} > 1$ represents the upsampling factor) on the SPS image prior to its processing with the NCPP algorithm. This approach can effectively enlarge the object domain size by a factor of $\alpha$, resulting in a new dimension of $\alpha N\Delta {x}_{0}$, thus ensuring adequate coverage of the FOV. It is essential to select the upsampling factor $\alpha$ to be as small as possible, i.e., larger than but close to ${D}_{{\rm{FOV}}}/N\Delta {x}_{0}$, since a too large $\alpha$ may compromise the quality of the reconstruction. For Fig. 3g, the raw SPS image (2048 × 2048) was upsampled by a factor of 1.25 to 2560 × 2560 using bilinear interpolation. For Fig. 3k, I, the raw SPS images (1024 × 1024) were upsampled by a factor of 2 to 2048 × 2048 through bilinear interpolation.

### Phase retrieval algorithm

The NCPP algorithm (Algorithm 1 in Supplementary Section 3) was designed to address the reconstructions from Fraunhofer-diffraction (far-field approximation) SPS pattern (Figs. 4e–k and 5). It combines the ping-pong algorithm with a set of constraints involving decreasing nonlinear moduli^40^ in the Fourier frequency domain. This approach allows for the full utilization of low-SNR high-frequency components and enhances the tolerance for overexposure and low dynamic range of the SPS patterns. Notably, no support constraint was imposed in the spatial domain. The phase-modulated NCPP algorithm (Algorithm 2 in Supplementary Section 4) was developed for the reconstructions of Fresnel-diffraction (paraxial approximation) SPS images (Fig. 2, Fig. 3, and Fig. 4b, m, o). The spherical phase term$\Psi$ in Fresnel diffraction integral was initially estimated using the pre-measured object-to-screen distance *Z*_OS_ and the calibrated object-plane sampling interval $\Delta {x}_{0}$. Afterward, as a known phase modulation, the spherical phase term $\varPsi$, along with a support constraint^38,50^ derived from the SPS pattern or estimated roughly, was imposed on the spatial domain in the iteration. This process allows us to reconstruct the object images from the captured Fresnel SPS patterns.

## Supplementary information

Supplementary Information for Relay-projection microscopic telescopy

Supplementary Video for Relay-projection microscopic telescopy (rPMT)

## Data Availability

The data that support the findings of this study are available from the corresponding authors upon reasonable request.
