# Supplementary material for: Relay-projection microscopic telescopy
Source: Light Sci Appl. 2025 Mar 7;14:117. doi: 10.1038/s41377-025-01800-6 (PMC11885427; doi:10.1038/s41377-025-01800-6)
Supplement: Supplementary file 1 — Supplementary Information for Relay-projection microscopic telescopy [file 41377_2025_1800_MOESM1_ESM.docx]

Supplementary Information for

**Relay-projection microscopic telescopy**

Wenjun Yi^1,^*^,§^, Shuyue Zhu^1,2,*^, Meicheng Fu^1^, Nan Gu^1^, Junli Qi^1^, Siyu Liu^1^, Mengjun Zhu^1,3^, Ping Wang^4^, Xin Chen^1^, Yi Zhang^1^, Hongyu Zhang^1^, Yao Xu^1^, Junyi Du^5^, Peng Xiong^5^, Zhaohua Dong^5^, Luobing Dong^5^, Qiong Liu^6,§^, and Xiujian Li^1,§^

*^1^College of Science,* *National University of Defense Technology, 410073, Changsha, China*

*^2^ Xi’an Satellite Control Center, Xi’an, 710043, China*

*^3^School of Information and Electronics Engineering, Hunan City University, 413000, Yiyang, China*

*^4^College of Electronic Science, National University of Defense Technology, 410073, Changsha, China*

*^5^ The School of Computer Science and Technology, Xidian University, Xi’an, 710071, China*

*^6^ Senior Department of Otolaryngology Head and Neck Surgery, the 6th Medical Center of Chinese PLA General Hospital, Chinese PLA Medical School, Beijing, 100048, China*

*^*^ These authors contributed equally to this paper*

*^§^ Correspondence: Wenjun Yi, Email: yiwenjun@nudt.edu.cn; Qiong Liu, Email: joanlq@sina.com; Xiujian Li, Email:* *[xjli@nudt.edu.cn](mailto:8xjli@nudt.edu.cn)*

This supplemental document contains the following information:

1. Comparison of direct and indirect light collection configurations

2. Abbe resolution limit and simulation analyses

3. Nonlinear-constraint ping-pong phase retrieval algorithm

4. Phase-modulated NCPP algorithm

5. Phase contrast imaging tests of rPMT

6. Analysis of rPMT through scattering media

7. Long-distance rPMT experiments with a smartphone

8. Random-matrix encoding masks used for Fig. 2f

9. Handmade reflective objects for long-distance rPMT

10. Homography-based distortion correction

11. Runtimes under various conditions

12. Robustness tests of rPMT

13. Signal to noise ratio analysis

Supplementary Video:

1. Reconstructed video through rPMT

**1. Comparison of direct and indirect light collection configurations**


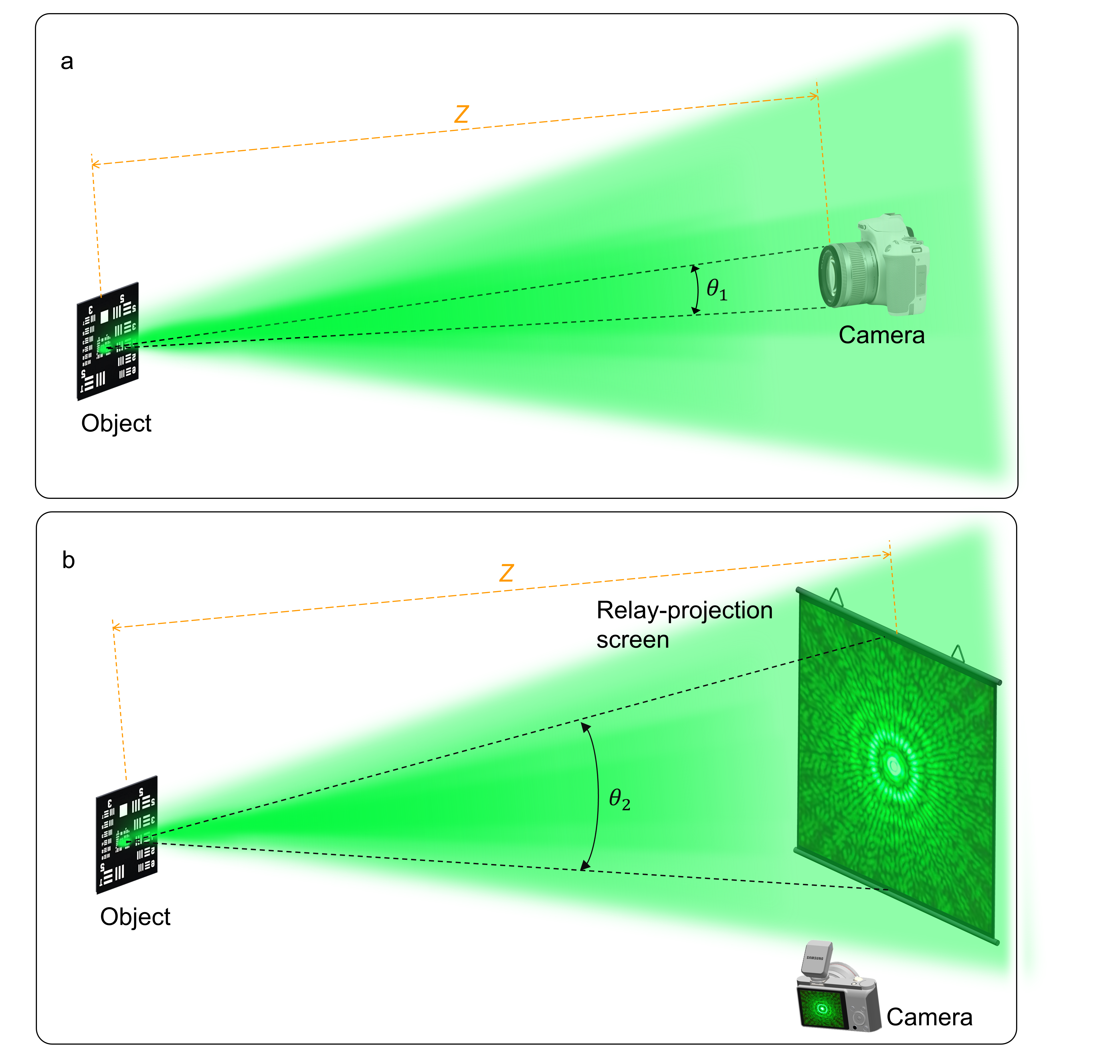


**Fig. S1. Direct and indirect light collection configuration. a,** direct (line-of-sight) light collection configuration. **b,** indirect (non-line-of-sight) light collection configuration.

In direct light collection configurations as shown in Fig. S1a, the camera must directly face and focus on the object to collect light from the objects. In this work, the relay-projection microscopic telescopy (rPMT) utilizes indirect light collection with the additional square-law transform introduced by diffuse reflection of the relay-projection screen, as illustrated in Fig. S1b. This process involves projecting the object's spatial power spectrum (SPS) pattern on a relay screen (regarded as the first square-law light collection since the surface diffuse reflection causes an equal probability of photon reflection in all directions) and then using a camera to capture the projected SPS (regarded as the second square-law light collection), in a similar manner to watching a movie by focusing our eyes on the diffusing screen rather than the slide films. It should be pointed out that, the distance between the object and the relay-projection screen is flexible and encompasses a broad range.

The fundamental difference between the direct light collection configurations and the indirect configurations hinges on their respective methodologies. In a direct configuration, the process involves a single square-law light collection on the sensor based on coherent or incoherent superposition of line-of-sight light, either directly or facilitated by lenses and reflectors. Conversely, in an indirect configuration, the process can be conceptualized as a non-line-of-sight light collection process, which unfolds in two distinct steps: the first square-law relay-projection on the screen and the second square-law light collection on the sensor. This differentiation holds significant importance, as it profoundly influences the primary determinant governing imaging resolution capability.

**2.** **Abbe resolution limit and simulation analyses**

Illuminated by a coherent monochromatic plane wave of wavelength$\lambda$, an object’s diffraction intensity distribution at a propagation distance of *Z* under the Fresnel (paraxial) approximation can be described^1^ as

(S1)

where $U\left(x_{0},y_{0}\right)$ and $U_{Z}\left(x,y\right)$ respectively represent the wavefield distribution in the object plane $\left(x_{0},y_{0}\right)$ and the observation plane $\left(x,y\right)$; denotes a two-dimensional (2D) Fourier transform operator with the spatial-frequency coordinates $\left(f_{x},f_{y}\right)$; the wavenumber *k* is defined as $k=2\pi/\lambda$. In a direct lensless coherent diffraction imaging system, *Z* represents the distance between the object and the detector. In contrast, in our rPMT system, *Z* is defined as the object-to-screen distance *Z*_OS_.

Furthermore, under the Fraunhofer (far-field) approximation, i.e.,$Z>>k\left(x_{0}^{2}+y_{0}^{2}\right)/2$, the diffraction intensity distribution in the observation plane, namely the spatial power spectrum (SPS), can be simplified as

(S2)

According to Fourier optics theory, the low-resolution features of objects correspond to the low spatial-frequency components, which are located around the origin (the zero frequency) in the frequency domain; meanwhile, the high-resolution features correspond to the high spatial-frequency components, which are located on the periphery, far from the frequency-domain origin. Therefore, the relationship$f_{x}=\frac{x}{\lambda Z},f_{y}=\frac{y}{\lambda Z}$ between the observation-plane coordinates$\left(x,y\right)$ and the frequency-domain coordinates$\left(f_{x},f_{y}\right)$ in Eqs. S1-S2 implies that, a finite-sized observation plane with a side length of *L* results in a finite spatial-frequency bandwidth. In other words, a single finite observation plane, such as the entrance pupil of a camera lens or the imaging sensor array of a lensless imaging system, acts as a low-pass filter and limits the resolution of the imaging system. At this point, taking into account $-L/2\leq x,y\leq L/2$ in the observation plane, the cutoff spatial-frequency is determined by$f_{x,\text{cut}}=\frac{L}{2\lambda Z},f_{y,\text{cut}}=\frac{L}{2\lambda Z}$. This implies that for a fixed-size observation plane, a longer propagation distance *Z* results in a lower cutoff frequency, which leads to resolution degradation. In fact, the best-resolved features corresponding to the exact cutoff frequency satisfy a resolution capability of $2\lambda Z/L$, known as the Abbe resolution limit, which degrades with the increase of the observation distance *Z*.

In this view, the fundamental problem of resolution improvement is the limitation of the receive aperture’s size. Apart from the adoption of a larger single aperture with high cost, most current approaches on resolution improvement involve the concept of synthetic receive aperture, such as synthetic aperture ladar and ptychographic imaging. These techniques typically shift the aperture perpendicular to the propagation direction to perform multiframe measurements, with the goal of achieving a significantly larger equivalent aperture compared to using a single finite aperture.

The proposed relay-projection microscopic telescopy (rPMT) fundamentally changes the method of light collection from objects, which can realize a high-resolution capability for ultra-wide working distance microscopic imaging and long-distance imaging from a single-shot capture. Compared with current super-resolution imaging techniques, there are no requirements for synthetic aperture, ptychography scanning or wavefront modulation. Our key insight is to significantly broaden the spatial-frequency bandwidth of an imaging system through square-law relay projection and non-line-of-sight light collection, rather than synthetic aperture.

**Fig. S2. Simulated SPS images along with the corresponding reconstructions at various distances. a,** a discretized USAF 1951 resolution target used in the simulations. **b,** the detailed region of **a**. **c1-c4,** The normalized SPS patterns recorded at 0.5 m, 1 m, 2 m and 3 m, respectively. **d1-d4,** The corresponding reconstructed objects by inverse propagation.

**Table S1. Line width (µm) in USAF Target 1951**

| Group  Element | 2 | 3 | 4 | 5 | 6 | 7 |
| --- | --- | --- | --- | --- | --- | --- |
| 1 | 125.00 | 62.50 | 31.25 | 15.63 | 7.81 | 3.91 |
| 2 | 111.36 | 55.68 | 27.84 | 13.92 | 6.96 | 3.48 |
| 3 | 99.21 | 49.61 | 24.80 | 12.40 | 6.20 | 3.10 |
| 4 | 88.39 | 44.19 | 22.10 | 11.05 | 5.52 | 2.76 |
| 5 | 78.75 | 39.37 | 19.69 | 9.84 | 4.92 | 2.46 |
| 6 | 70.15 | 35.08 | 17.54 | 8.77 | 4.38 | 2.19 |

Based on the angular spectrum theory^1^, we conducted a series of analyses on the effects of the imaging distance and receiving aperture through numerical simulations. The SPS images in Figs. S2-S4 are derived from numerical simulations based on angular spectrum theory, rather than from camera acquisitions. The SPS images are generated through angular spectrum propagation, which offers enhanced accuracy compared to the Fresnel diffraction formula, as the latter is a paraxial approximation of the former.

Taking a discretized version of a USAF target as the transmissive object, as shown in Fig. S2a, the total sampling number is set to 2340×2340 with a sampling interval of 5.95 µm, which ensures that the object’s line widths are consistent with those of a standard USAF 1951 resolution target (as listed in Table S1). The side lengths of the object plane and the observation plane are both $L=13.93\text{ mm}$. When illuminated by a coherent monochromatic plane wave of 532 nm, the object’s diffraction field distribution at a propagation distance of *Z* ($Z=0.50\text{ }m,1.00\text{ }m,2.00\text{ }m,3.00\text{ }m$) can be recorded using a fixed sensing area (receiving aperture) of $L\times L$. As shown in Fig. S2c1-c4, the actual size of the diffraction pattern increases as the imaging distance *Z* increases; consequently, a fixed receiving aperture will capture a diffraction field with a lower cutoff frequency, resulting in a degradation of resolution, as illustrated in Fig. S2d1-d4.

It should be noted that, instead of using a specific phase retrieval (PR) algorithm, the reconstructions here are directly achieved by performing inverse angular spectrum propagation on the recorded complex field that contains the phase information. This aims to eliminate the effects introduced by different PR algorithms and algorithm parameters. Given that PR algorithms operate solely on the field modulus without the phase information, the results obtained through the inverse propagation of the complex field are expected to be superior to those through PR reconstructions.

In more details, the Abbe diffraction-limited line width corresponding to the diffraction field in Fig. S2c1 can be calculated as $\delta_{\text{lw}}(Z=\text{0.50 m})=\lambda Z/L=\text{19.10 μm}$. It can be observed that the resolvable line width of the corresponding inverse-propagation reconstruction (Fig. S2d1) is indeed between group 4, element 5 (G4E5 for short) with a line width of 19.69 µm and G4E6 with a line width of 17.54 µm. Similarly, the diffraction-limited line width corresponding to Fig. S2c2,d2 is $\delta_{\text{lw}}(Z=\text{1.00 m})=\text{38.19 μm}$, between G3E5 (39.37 µm) and G3E6 (35.08 µm); the best-resolved line width corresponding to Fig. S2c3,d3 is $\delta_{\text{lw}}(Z=\text{2.00 m})=\text{76.38 μm}$, between G2E5 (78.75 µm) and G2E6 (70.15 µm); the diffraction-limited line width corresponding to Fig. S2c4,d4 is $\delta_{\text{lw}}(Z=\text{3.00 m})=\text{114.57 μm}$, between G2E1 (125.00 µm) and G2E2 (111.36 µm). The simulation results demonstrate that a single finite receive aperture will limit the cutoff frequency, thereby reducing the resolution capability in long-distance imaging. The resolvable features align precisely with the Abbe diffraction limitation.

To illustrate the effects of coarser sampling resulting from a finite number and size of pixels, we examine two scenarios: (1) maintaining a constant cutoff frequency while varying both the size and quantity of pixels, and (2) varying the cutoff frequencies while keeping either the pixel size or the pixel count constant.

Fig. S3 presents the first case. Panels (a1-a4) display the normalized SPS patterns recorded at a distance of Z=0.5 m, sampled at varying intervals while maintaining a constant sensor array size, which results in a consistent cutoff frequency. Despite the cutoff frequencies remaining constant, the quality of the reconstructed images varies, as illustrated in panels (b1-b4) and (c1-c4). Specifically, under conditions of a fixed cutoff frequency, the resolution of the reconstructed images deteriorates as the sampling interval increases.


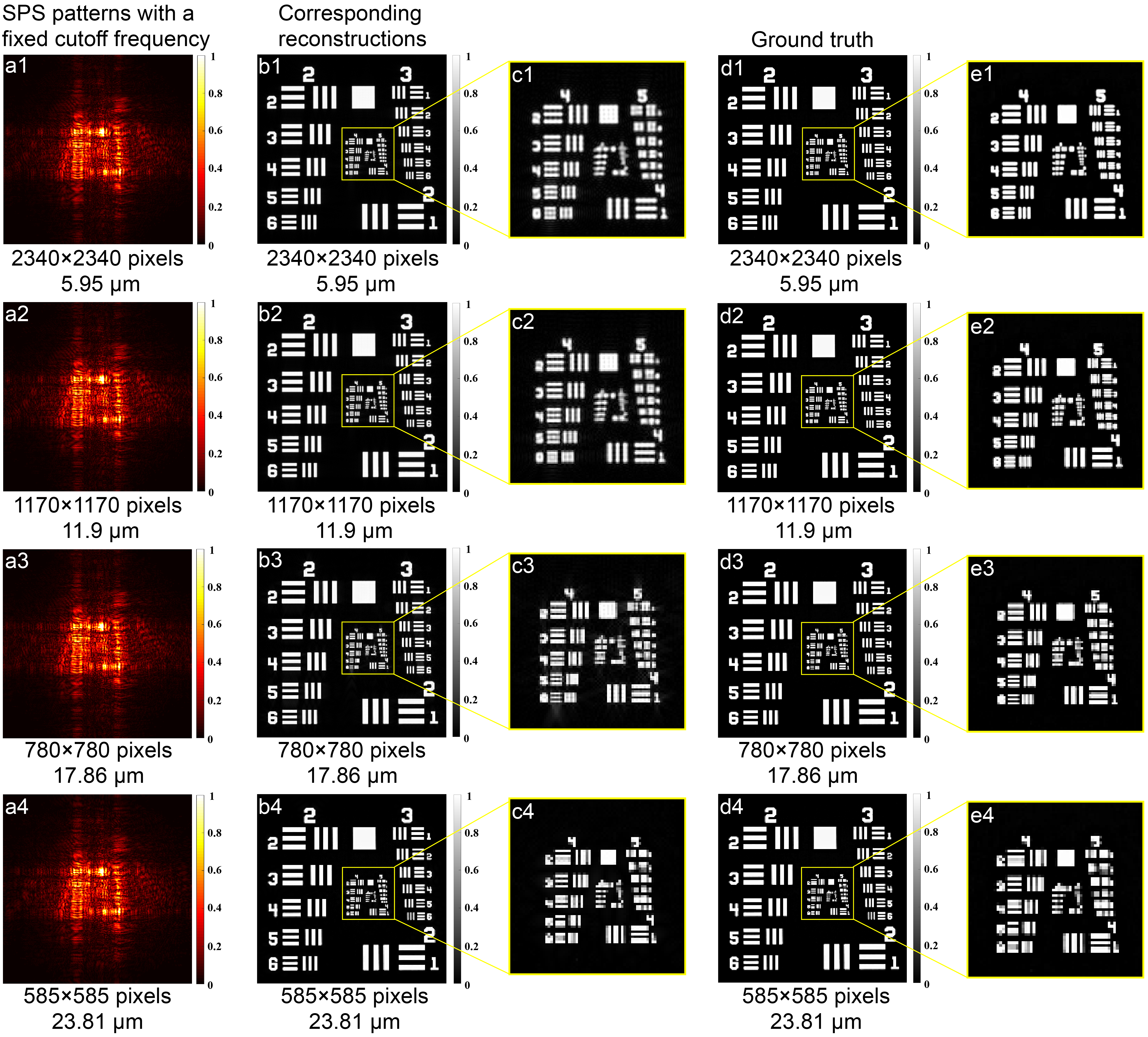


**Fig. S3.** **Simulated SPS images along with the corresponding reconstructions at various sampling intervals under a fixed cutoff frequency. a1-a4,** The normalized SPS patterns recorded at Z=0.5 m but different sampling intervals under the condition of a fixed cutoff frequency. **b1-b4,** The corresponding reconstructed objects by inverse propagation. **c1-c4,** Detailed region (Groups 4,5 of the USAF 1951 resolution target) cropped from **b1-b4**. **d1-d4,** The corresponding ground truths. **e1-e4,** Detailed region (Groups 4,5 of the USAF 1951 resolution target) cropped from **d1-d4**.

The simulated results of the second case are illustrated in Fig. S4. Panels (a1-a4) of Fig. S4 present the SPS patterns sampled at Z=0.5 m with a consistent sampling interval of 5.95 μm but varying sampling numbers, while panels (b1-b4) depict their corresponding reconstruction images. These images demonstrate a significant degradation in spatial resolution due to the reduction of the cutoff frequency, and the best-resolved line widths of the reconstructed images align closely with the Abbe diffraction limit. For instance, the Abbe diffraction-limited line width corresponding to Fig. S4a1 is calculated as 22.93 µm, while the resolvable line width of the corresponding reconstruction (Fig. S4b1) falls between G4E3 with a line width of 24.80 µm and G4E4 with a line width of 22.10 µm.

Conversely, panels (d1-d4) of Fig. S4 show SPS patterns sampled at Z=0.5 m with the same sampling number of 2340×2340 but different sampling intervals, and their respective reconstructions are displayed in panels (e1-e4). Similar to the previous set, these images also exhibit a significant decline in resolution due to the reduced cutoff frequency, with the resolvable line widths again closely aligning with the Abbe diffraction limit.

It is important to note that in Fig. S4 the cutoff frequency of panel (a*i*) (*i*=1,2,3,4) is identical to that of panel (d*i*). Consequently, this pair of SPS patterns, along with their corresponding reconstructions (panels (b*i*) and (e*i*)), exhibits significant visual similarities. However, upon magnifying small regions of these images, differences arising from varying sampling intervals become evident, as illustrated in Fig. S4a5 compared to d5 and b5 compared to e5.


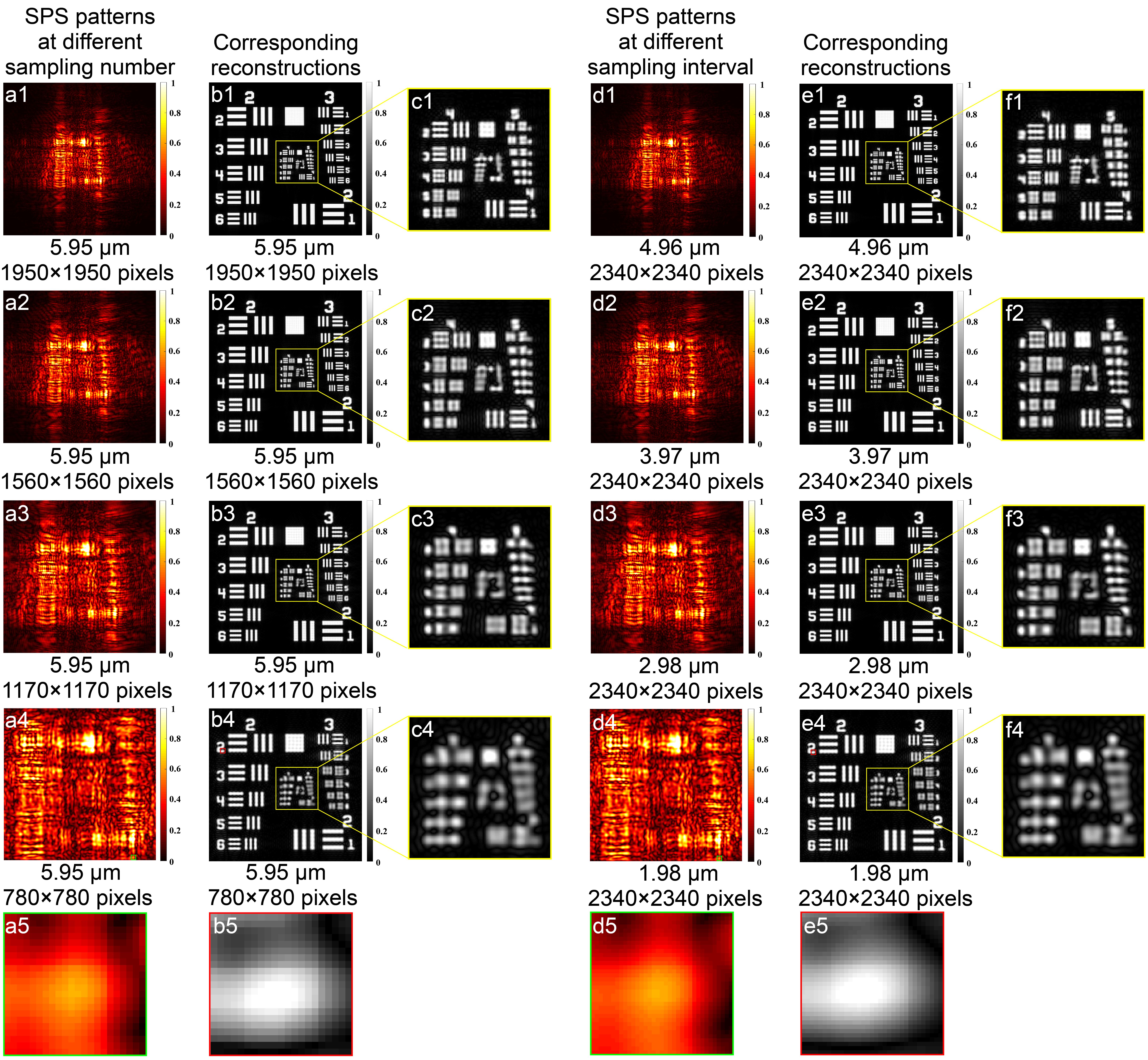


**Fig. S4. Simulated SPS images along with the corresponding reconstructions at different sampling intervals and sampling numbers across various cutoff frequencies. a1-a4,** The normalized SPS patterns recorded at Z=0.5 m with the same sampling interval of 5.95 μm but different sampling numbers. **b1-b4,** The corresponding reconstructed objects by inverse propagation from **a1-a4**. **c1-c4,** Detailed region cropped from **b1-b4**. **d1-d4,** The normalized SPS patterns recorded at Z=0.5 m with the same sampling number of 2340×2340 but different sampling intervals. **e1-e4,** The corresponding reconstructed objects by inverse propagation from **d1-d4**. **f1-f4,** Detailed region cropped from **e1-e4**. The cutoff frequencies of **a1-a4** are respectively identical to those of **d1-d4**. To illustrate the differences between SPS images in a row resulting at different sampling intervals, the corresponding small regions marked by green rectangles in **a4** and **d4** are magnified in **a5** and **e5**. Likewise, **b5** and **e5** depict the small regions associated with **b4** and **e4**, respectively.

**3.** **Nonlinear-constraint ping-pong phase retrieval algorithm**

Due to the degradations of the signal-to-noise ratio (SNR) and dynamic range of the SPS pattern captured by rPMT, basic PR algorithms, such as the error reduction algorithm (ER)^2^, hybrid-input output algorithm (HIO)^3^ and Ping-pong algorithm^4^, cannot effectively reconstruct the accurate object image from a single-shot SPS pattern. In order to retrieve the object image from a single SPS pattern with low SNR and dynamic range, we propose a specific PR algorithm called nonlinear-constraint Ping-pong algorithm (NCPP) as described below. The NCPP algorithm combines the Ping-pong algorithm with a series of variable nonlinear Fourier modulus constraints^5^. This combination allows for the full utilization of low-SNR high-frequency components to significantly enhance the tolerances for overexposure and low dynamic range in long-exposure SPS images. In detail, a larger nonlinear Fourier modulus is first utilized to extract the low-frequency components and enhance tolerance for overexposure in these components, aiding in the recovery of the object's outlines. Subsequently, a smaller nonlinear Fourier modulus is adopted to extract the high-frequency components, allowing for the retrieval of the object's fine structural details. Besides, the NCPP algorithm can effectively prevent the solution from getting stuck in local minima by alternating the object-domain constraints of ER and HIO. The nonlinear Fourier modulus constraints introduced in the frequency domain will also contribute to much faster convergence.

**Algorithm 1** Nonlinear constraint ping-pong (NCPP) phase retrieval algorithm

**Input:** Intensity-only measurement (far-field power spectrum) *I*,

Initial estimate of the object image$g_{0}$, and set iterative initial value $g^{(0)}=g_{0}$,

**Outer loop:**

**For** $\gamma=1.2\text{ to }0.8\text{ (in steps of -0.2)}$ **do**

Set the nonlinear Fourier modulus $M=\left(\sqrt{I}\right)^{\gamma}$

**Middle loop:**

**For** $\beta=\text{3 to 1 (in steps of -0.2)}$ **do**

**Inner loop:**

**For** $j=\text{0 to 30(in steps of 1)}$ **do**

1) Fourier transform: $G^{(j)}\leftarrow\text{FFT}\left\{g^{(j)}\right\}$

where FFT represents the fast Fourier transform algorithm and $G^{(j)}$ denotes the fast Fourier transform of $g^{(j)}$.

2) Keep current Fourier phase, but impose Fourier modulus constraint: $G^{(j)}\leftarrow M\cdot\frac{G^{(j)}}{\left|G^{(j)}\right|}$

3) Inverse Fourier transform: $g'^{(j)}\leftarrow\text{IFFT}\left\{G^{(j)}\right\}$

where IFFT represents the inverse fast Fourier transform algorithm, and $g'^{(j)}$ denotes the inverse fast Fourier transform of $G^{(j)}$, i.e., the updated object image.

4) Impose pointwise constraint in the object-domain:

$$g^{(j)}\leftarrow g'^{(j)}=\left\{\begin{matrix} g'^{(j)}\text{ }if\text{ }g'^{(j)}\geq0 \\ g^{(j)}-\beta\cdot g'^{(j)}\text{ }if\text{ }g'^{(j)}<0 \end{matrix}\right.$$

5) Fourier transform: $G^{(j)}\leftarrow\text{FFT}\left\{g^{(j)}\right\}$

6) Keep current Fourier phase, but impose Fourier modulus constraint: $G^{(j)}\leftarrow M\cdot\frac{G^{(j)}}{\left|G^{(j)}\right|}$

7) Inverse Fourier transform: $g'^{(j)}\leftarrow\text{IFFT}\left\{G^{(j)}\right\}$

8) Impose pointwise non-negative constraint in the object-domain:

$$g^{(j+1)}\leftarrow g'^{(j)}=\left\{\begin{matrix} g'^{(j)}\text{ }if\text{ }g'^{(j)}\geq0 \\ 0\text{ }if\text{ }g'^{(j)}<0 \end{matrix}\right.$$

**end**

**end**

**end**

**Output:** the reconstructed object image *g*

For comparison, four PR algorithms including ER, HIO, Ping-ping and NCPP were respectively performed on a single SPS image captured by the rPMT, and the reconstructions are summarized in Fig. S5. The ER algorithm and HIO algorithm almost completely failed to identify the target; although the Ping-pong algorithm can recover the rough outline of the target, the detailed structure, i.e., the high-frequency information, cannot be effectively retrieved from the low SNR and dynamic range SPS captured by the rPMT. Dramatically, the NCPP algorithm can achieve a high-fidelity reconstruction, retrieving the detailed structures of the target, such as the serif font features.


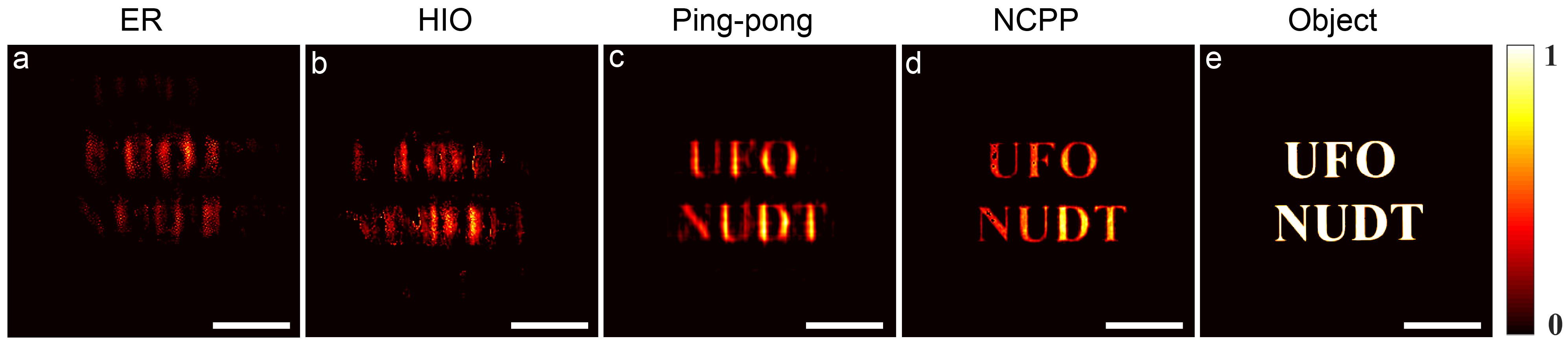


**Fig. S5 The results of four different PR algorithms from a single SPS image captured by rPMT. a**, The reconstruction of ER algorithm. **b**, The reconstruction of HIO algorithm. **c**, The reconstruction of Ping-pong algorithm. **d**, The reconstruction of NCPP algorithm. **e**, The ground truth. Scale bars: 1 mm.

**4. Phase-modulated NCPP algorithm**

According to Fourier optics theory, when illuminated by a coherent monochromatic wave of wavelength$\lambda$, the field distribution of an object’s Fresnel diffraction at a propagation distance of *Z* can be numerically calculated through a two-dimensional Fast Fourier Transform (FFT) as follows:

$\begin{matrix} U_{Z}\left(p\Delta x,q\Delta y\right)=\frac{\exp\left(jkZ\right)\exp\left(j\frac{k}{2Z}\left(\left(p\Delta x\right)^{2}+\left(q\Delta y\right)^{2}\right)\right)}{j\lambda Z}\cdot\\ FFT\left\{U_{0}\left(m\Delta x_{0},n\Delta y_{0}\right)\cdot\Psi_{\text{illu}}\left(m\Delta x_{0},n\Delta y_{0}\right)\cdot\exp\left[\frac{jk}{2Z}\left((m\Delta x_{0})^{2}+(n\Delta y_{0})^{2}\right)\right]\right\}\left|_{f_{x}=\frac{p\Delta x}{\lambda Z},f_{y}=\frac{q\Delta y}{\lambda Z}}\right. \end{matrix}$（S3）

where $U_{0}\left(m\Delta x_{0},n\Delta y_{0}\right)$ represents the discretized field distribution in the object plane with a sampling interval of $\Delta x_{0},\Delta y_{0}$ and a total sampling number of $M\times N$ ($-M/2\leq m\leq M/2,$ $-N/2\leq n\leq N/2$), while $U_{Z}\left(p\Delta x,q\Delta y\right)$ is the discretized diffraction field in the observation plane with a sampling interval of $\Delta x,\Delta y$ ($-M/2\leq p\leq M/2$,$-N/2\leq q\leq N/2$); the wavenumber *k* is defined as $k=2\pi/\lambda$. In a direct lensless coherent diffraction imaging system, *Z* represents the distance between the object and the detector. In contrast, in our rPMT system, *Z* is defined as the object-to-screen distance *Z*_OS_. The illuminating field in the object plane, denoted as $\Psi_{\text{illu}}\left(m\Delta x_{0},n\Delta y_{0}\right)$, varies depending on the type of illuminating wave. Specifically, for coherent planar waves, $\Psi_{\text{illu}}\left(m\Delta x_{0},n\Delta y_{0}\right)$ can be set to 1, whereas for coherent spherical waves, $\Psi_{\text{illu}}\left(m\Delta x_{0},n\Delta y_{0}\right)=\exp\left[\frac{jk}{2R_{\text{illu}}}\left((m\Delta x_{0})^{2}+(n\Delta y_{0})^{2}\right)\right]$, where $R_{\text{illu}}$ represents the curvature radius of the spherical waves in the object plane. According to the theories of the discrete Fourier transform and Nyquist sampling theorem^6^, the relationship between the frequency-domain coordinates $\left(f_{x},f_{y}\right)$ and the object-plane coordinates can be expressed as$f_{x}=\frac{p}{M\Delta x_{0}},$ $f_{y}=\frac{q}{N\Delta y_{0}}$. Consequently, the sampling intervals of the object plane and the observation plane can be linked by: $\Delta x=\frac{\lambda Z}{M\Delta x_{0}},\Delta y=\frac{\lambda Z}{N\Delta y_{0}}$.

According to Eq. S3, the objects cannot be directly retrieved from the captured Fresnel-diffraction SPS images using the NCPP algorithm proposed in Supplementary Section 3, because of the spherical phase term $\Psi(Z)=\exp\left[\frac{jk}{2Z}\left((m\Delta x_{0})^{2}+(n\Delta y_{0})^{2}\right)\right]$ and the illuminating field $\Psi_{\text{illu}}$ in the integrand of Eq. S3. To address this concern, the spherical phase term $\Psi(Z)$, along with the illuminating field $\Psi_{\text{illu}}$, can be combined with the original object $U_{0}\left(m\Delta x_{0},n\Delta y_{0}\right)$ to yield a composite object expressed as $U_{\text{c}}=U_{0}\cdot\Psi(Z)\cdot\Psi_{\text{illu}}$. Therefore, Fresnel diffraction can be viewed as a phase-modulated Fraunhofer diffraction, and the Fresnel SPS of the original object $U_{0}$ can be treated as the far-field SPS of the equivalent object $U_{\text{c}}$. Consequently, by imposing a measurable spherical-phase-modulation $\Psi(Z)$ and an illuminating field $\Psi_{\text{illu}}$on the object $U_{0}$, we further propose a phase-modulated NCPP algorithm that enables us to retrieve the objects from the Fresnel SPS captured by our rPMT.

An object image can be retrieved from its Fresnel-diffraction SPS pattern through the following phase-modulated NCPP phase retrieval algorithm. Compared with the NCPP algorithm for far-field SPS patterns, the phase-modulated NCPP algorithm introduces an illuminating field $\Psi_{\text{illu}}$, a spherical phase modulation in the FFT integrand ($\Psi_{0}(Z)$ in the object plane) and a spherical-phase prefactor to the integral ($\Psi_{i}(Z)$ in the observation plane). By inputting a random real matrix $g_{0}$ as the initial estimate$g^{(0)}$, one can perform the following phase-modulated NCPP on a SPS image. Simultaneously, the object-domain support constraint can be imposed in each iteration, resulting in the retrieval of a high-fidelity object image located within the support^2, 7^.

**Algorithm 2** phase-modulated NCPP phase retrieval algorithm

**Input:** Intensity-only measurement (Fresnel-diffraction power spectrum) *I*,

Initial estimate of the object image $g_{0}$, and set iterative initial value $g^{(0)}=g_{0}$,

Illuminating wavelength $\lambda$, object-screen distance *Z*_OS_, sampling number in a row *N*,

Sampling interval in the observation plane along the $x$ and $y$ axes $\Delta x$ and $\Delta y=\Delta x$, respectively , curvature radius $R_{\text{illu}}$ of illuminating wave in the object plane ( $R_{\text{illu}}$ can be set to $\infty$, i.e., $1/R_{\text{illu}}=0$, for the planar wave illumination),

Set the sampling interval in the object plane along the $x_{0}$ and $y_{0}$ axes $\Delta x_{0}=\frac{\lambda Z_{\text{OS}}}{N\Delta x}$ and$\Delta y_{0}=\Delta x_{0}$, respectively.

Set the phase term in the object plane$\Psi_{0}(Z_{\text{OS}})=\exp\left[\frac{i\pi}{\lambda}\left(\frac{1}{R_{\text{illu}}}+\frac{1}{Z_{\text{OS}}}\right)\left((p\Delta x_{0})^{2}+(q\Delta y_{0})^{2}\right)\right]$,

Set the phase term in the observation plane $\Psi_{i}(Z_{\text{OS}})=\exp\left[\frac{i\pi}{\lambda Z_{\text{OS}}}\left((p\Delta x)^{2}+(q\Delta y)^{2}\right)\right]$,

where *p* and *q* are integers that satisfy$-N/2\leq p,q\leq N/2$.

**Outer loop:**

**For** $\gamma=1.2\text{ to }0.8\text{ (in steps of -0.2)}$ **do**

Set the nonlinear Fourier modulus to be $M=\left(\sqrt{I}\right)^{\gamma}$

**Middle loop:**

**For** $\beta=\text{3 to 1 (in steps of -0.2)}$ **do**

**Inner loop:**

**For** $j=\text{0 to 30 (in steps of 1)}$ **do**

1) Fresnel-diffraction forward propagation: $G^{(j)}\leftarrow\Psi_{i}(Z_{\text{OS}})\cdot\text{FFT}\left\{g^{(j)}\cdot\Psi_{0}(Z_{\text{OS}})\right\}$

where FFT represents the fast Fourier transform algorithm and $G^{(j)}$ denotes the complex-amplitude distribution of the Fresnel diffraction field at an object-screen distance of *Z*_OS_.

2) Keep current Fourier phase, but impose Fourier modulus constraint: $G^{(j)}\leftarrow M\cdot\frac{G^{(j)}}{\left|G^{(j)}\right|}$

3) Fresnel-diffraction backward propagation:

$$g'^{(j)}\leftarrow\text{conj}\left[\Psi_{0}(Z_{\text{OS}})\right]\cdot\text{IFFT}\left\{G^{(j)}\cdot\text{conj}\left[\Psi_{i}(Z_{\text{OS}})\right]\right\}$$

where IFFT represents the inverse fast Fourier transform algorithm, $\text{conj}\left[\cdot\right]$ denotes the complex conjugate operator, while $g'^{(j)}$ denotes the Fresnel backward propagation of $G^{(j)}$, i.e., the updated object image.

4) Impose support constraint and/or the following pointwise constraint in the object-domain:

$$g^{(j)}\leftarrow g'^{(j)}=\left\{\begin{matrix} g'^{(j)}\text{ }if\text{ }g'^{(j)}\geq0 \\ g^{(j)}-\beta\cdot g'^{(j)}\text{ }if\text{ }g'^{(j)}<0 \end{matrix}\right.$$

5) Fresnel-diffraction forward propagation: $G^{(j)}\leftarrow\Psi_{i}(Z_{\text{OS}})\cdot\text{FFT}\left\{g^{(j)}\cdot\Psi_{0}(Z_{\text{OS}})\right\}$

6) Keep current Fourier phase, but impose Fourier modulus constraint: $G^{(j)}\leftarrow M\cdot\frac{G^{(j)}}{\left|G^{(j)}\right|}$

7) Fresnel-diffraction backward propagation:

$$g'^{(j)}\leftarrow\mathrm{conj}\left[\Psi_{0}(Z_{\text{OS}})\right]\cdot\text{IFFT}\left\{G^{(j)}\cdot\text{conj}\left[\Psi_{i}(Z_{\text{OS}})\right]\right\}$$

8) Impose support constraint and/or the following pointwise non-negative constraint in the object-domain:

$$g^{(j+1)}\leftarrow g'^{(j)}=\left\{\begin{matrix} g'^{(j)}\text{ }if\text{ }g'^{(j)}\geq0 \\ 0\text{ }if\text{ }g'^{(j)}<0 \end{matrix}\right.$$

**end**

**end**

**end**

**Output:** the reconstructed object image *g*

**5. Phase contrast imaging tests of rPMT**

To evaluate the rPMT’s capability in imaging phase objects, we conducted a series of experiments using phase objects generated by a reflective-mode phase-only Spatial Light Modulator (SLM, HOLOEYE PLUTO, 1920×1080 pixels, 8 µm pixel pitch size, 8-bit grey levels). As depicted in Fig. S6a, various images (Figs. S6c, g, and i) were loaded onto the phase-only SLM to serve as phase objects, which were illuminated by a coherent light source. Subsequently, the SPS patterns were projected onto a relay screen and captured by a 4-megapixel monochrome camera equipped with a fixed-focus imaging lens of a 25 mm front aperture.

In these experiments, the object-screen distance was set to *Z*_OS_ = 957.9 mm, and the camera lens was positioned 965.0 mm away from the relay screen. The phase contrast image shown in Fig. S6d was retrieved using the NCPP algorithm from the single-shot SPS pattern depicted in Fig. S6b, with a detailed region illustrated in Fig. S6f. When compared to the loaded target (Fig. S6c), it is evident that the phase object is faithfully reconstructed with high resolution. Additional results, presented in Figs. S6g-j, further support this conclusion.


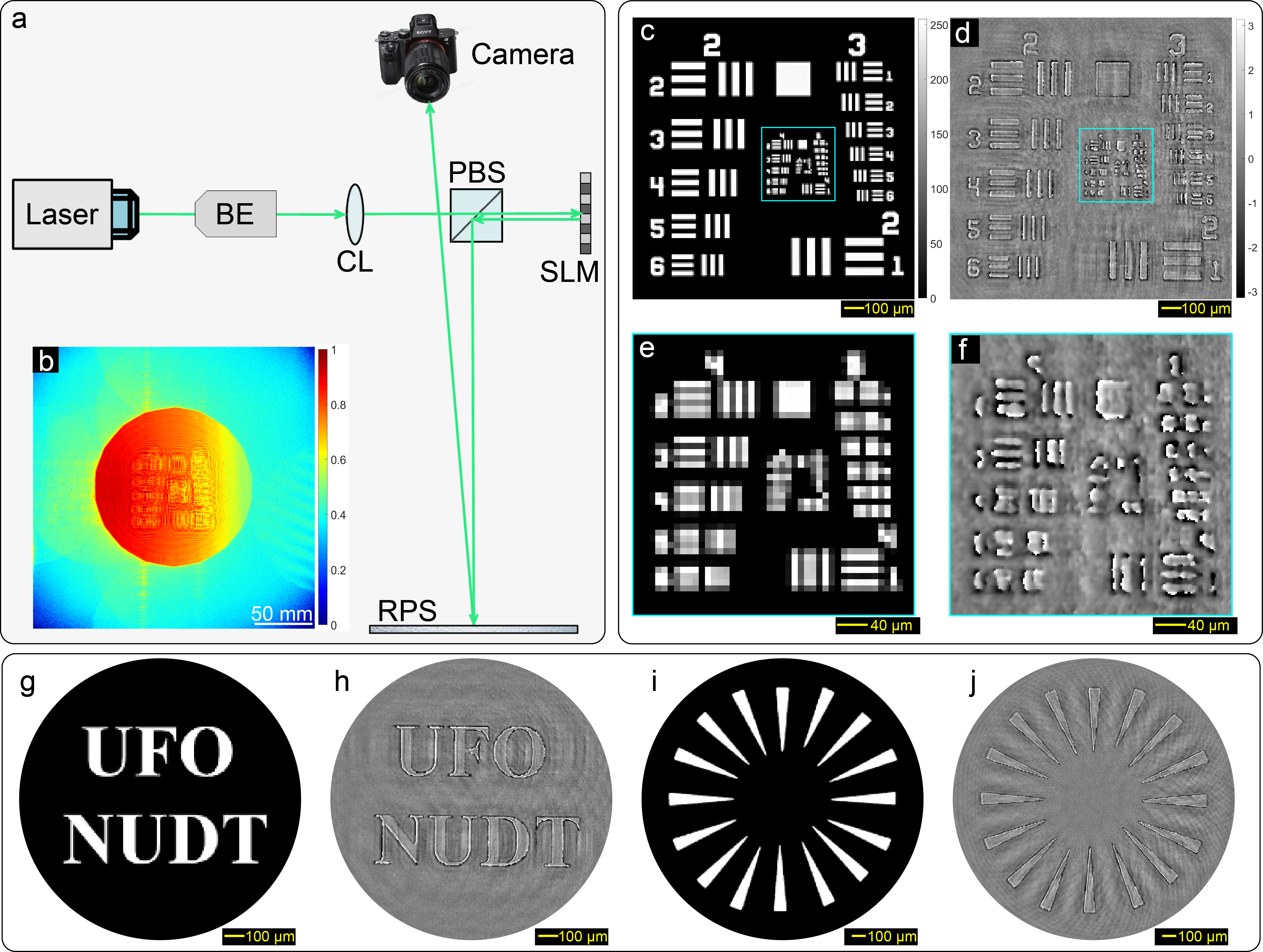


**Fig. S6.** **Phase contrast imaging tests** **of rPMT at a projection distance of *Z*_OS_*=*957.9 mm. a**, The experimental setup: BE, beam expander; CL, convergent lens; PBS, polarized beam splitter; RPS, relay-projection screen. **b,** The normalized SPS image corresponding to **c**. **c**, The resolution target image loaded into the phase-only SLM, whose line widths are not identical with those of a standard USAF 1951 target. **d**, The retrieved single-shot phase contrast image from **b**. **e** and **f**, The corresponding detailed regions of **c** and **d**. **g** and **i**, Another two loaded images. **h** and **j,** the corresponding retrieved phase contrast images for **g** and **i**.


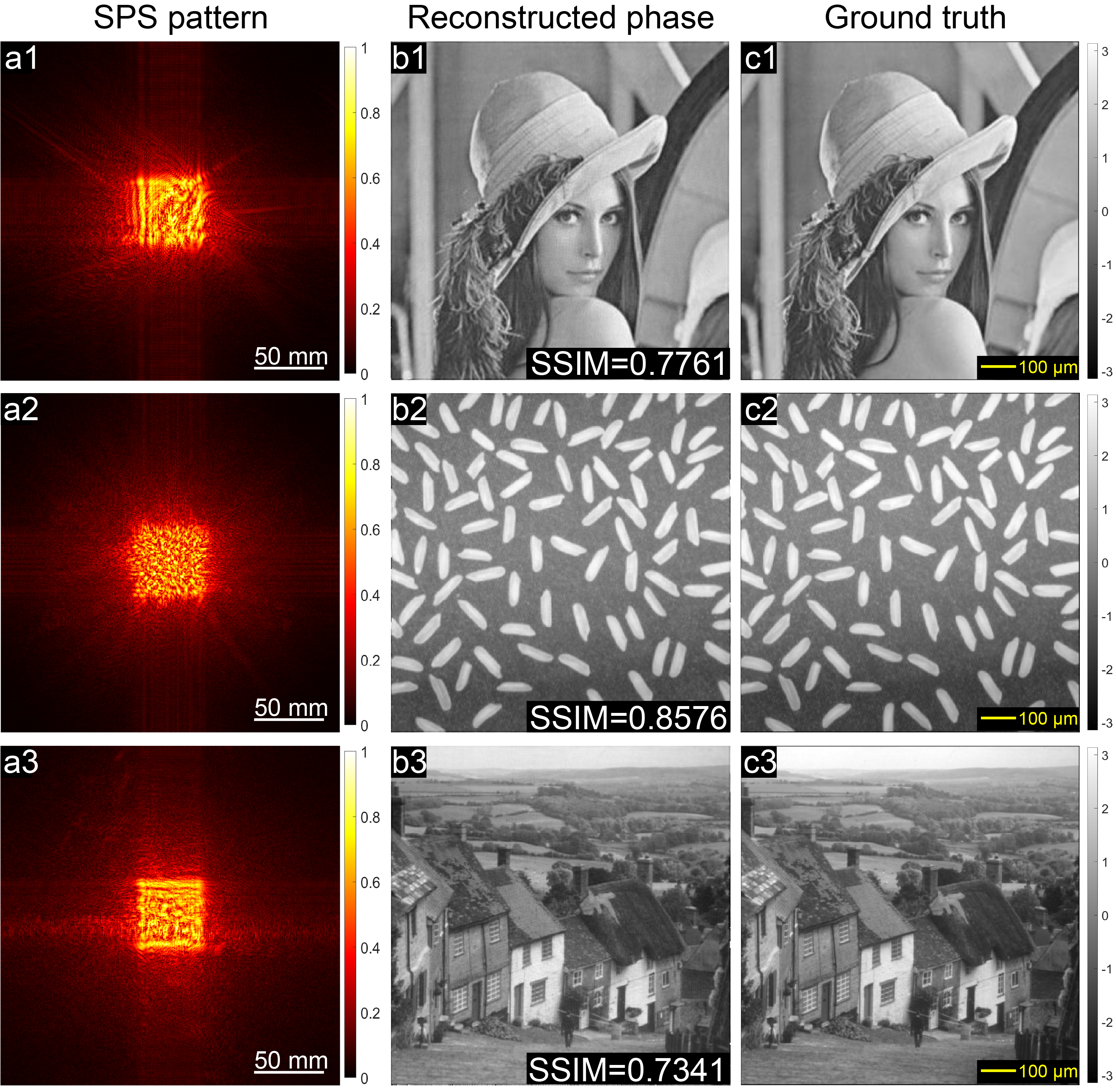


**Fig. S7. Simulational rPMT experiments under the same conditions with rPMT experiments on the phase-only SLM shown in Fig. 4. a1-a3,** The normalized SPS images images. **b1-b3,** Reconstructed phase objects from **a1-a3**, respectively. **c1-c3,** The ground truths.

To conduct quantitative assessments of phase reconstructions, we performed a series of simulations under conditions identical to those used in the rPMT experiments with the phase-only SLM. These conditions included the object-to-screen distance, the sampling interval on the observation plane, and the effective size of the relay screen. The reconstructed phase objects from SPS images in Fig. S7a1-a3 are presented in Fig. S7b1-b3. Despite some degree of distortion, the SSIM values between the reconstructed phases and the actual phases indicate satisfactory results.

**6. Analysis of rPMT through scattering media**

Under the constraints of the optical memory effect, the scattered single-pixel spectrum (SPS) of an object is essentially identical to its scatter-free counterpart, as described in our previous work^8^. This relationship can be derived concisely as follows. Under coherent illumination, the light from an object propagates a distance to a diffuser. If the object size, the object-diffuser distance and the scattering layer thickness satisfy the memory-effect constraint^9-11^, the diffraction light field passing through the diffuser can be represented by $u=u_{0}*h$, where $u_{0}$ is the object field and the symbol denotes a convolution operation; $h$ is the point-spread function (PSF) induced by the diffuser, which remains translation-invariant due to the optical memory effect. The PSF’s autocorrelation $h★h$ is a sharply peaked function $c_{1}\delta+c_{2}$, in which $\delta$ is the two-dimensional Dirac function,$c_{1}$ is a constant and $c_{2}$is the PSF’s autocorrelation background term^12^. Therefore, the autocorrelation of *u* can be expressed as:

$\begin{matrix} u★u=\left(u_{0}*h\right)★\left(u_{0}*h\right)=\left(u_{0}★u_{0}\right)*\left(h★h\right) \\ =\left(u_{0}★u_{0}\right)*\left(c_{1}\delta+c_{2}\right)=c_{1}\left(u_{0}★u_{0}\right)+c_{2}*\left(u_{0}★u_{0}\right) \end{matrix}$ (S4)

The scattered far-field SPS can be achieved by performing a Fourier transform on Eq. S4 and be rewritten as follows according to the Wiener-Khinchin theorem,

(S5)

Generally, $c_{2}$can be treated as an approximate constant, and thus the second term in Eq. S5 can be rewritten as , which only affects the zero frequency of the SPS and has no impact on phase retrieval reconstructions. Eq. S5 suggests the object’s scattered far-field SPS, i.e., , is identical to its scatter-free far-field diffraction SPS , which implies that we can reveal the image of an object behind a scattering layer by directly capturing the scattered SPS and performing a phase retrieval reconstruction. Furthermore, to enable long-distance scattering imaging, herein we utilize rPMT to capture a large-scale long-distance SPS pattern, and retrieve the object image from the low SNR and dynamic range SPS pattern by the proposed NCPP algorithm.

It should be noted that in practical measurements, only the low and middle frequency components of the scattered SPS turn out to be consistent with those of scatter-free SPS as shown in Fig. 5 in the main text, owing to the fact that $c_{2}$is actually subject to a randomly fluctuating spatial distribution, rather than a constant one. Therefore, the second term in Eq. S5 represents an inhomogeneous background imposed on the SPS, which will consequently obscure the low-SNR high-frequency information.

**7. Long-distance rPMT experiments with a smartphone**

As shown in Fig. S9a1-a2, the diffracted light of the transmissive objects shined onto a relay screen at a projection distance of *Z*_OS_ *=*96.0 m, and then the diffusely reflected light was collected by a smartphone (Huawei Mate20 with a LEICA camera, 12 megapixels, 8 bit dynamic range, a wide-angle lens of F/1.8 and an entrance aperture of 2 mm). The ISO was set to 100. To avoid the influences of multiple lenses on imaging resolution, we activate only the main lens by covering the others, as shown in Fig. S8.

The SPS patterns captured by the smartphone are listed in Fig. S9b1-b4, and the single-shot reconstructions by the NCPP algorithm are summarized in Fig. S9c1-c4. Obviously, the SSIMs of the reconstructions are all greater than 0.82. The SPS images captured with the smartphone have lower SNR and a smaller dynamic range (8 bit), due to the small entrance aperture (2 mm), the errors induced by the Bayer color filter, the JPEG compression, and the image conversion from RGB to grayscale. Despite all these limitations, the rPMT still performs satisfactorily and achieves high resolution.

The actual side length of the SPS pattern in Fig. S6b1 is about 1912.7 mm, and the line width of the recovered line pairs in Fig. S9c1 is 70.15 µm. Basically, at a projection distance of *Z*_OS_ *=*96.0 m, the best resolvable line width corresponding to a receive aperture of 1912.7 mm is 26.70 µm, while it’s 25.54 mm for the smartphone camera lens aperture of 2 mm. Therefore, the resolution limit of rPMT can theoretically be improved by 956 times compared with the phone camera used according to the spatial bandwidth enlargement. The practical resolution improvement is more than 364, which is lower than the theoretical value, due to the degradations of SNR and dynamic range. Certainly, rPMT can significantly reduce the hardware requirements of a super-resolution imaging system.


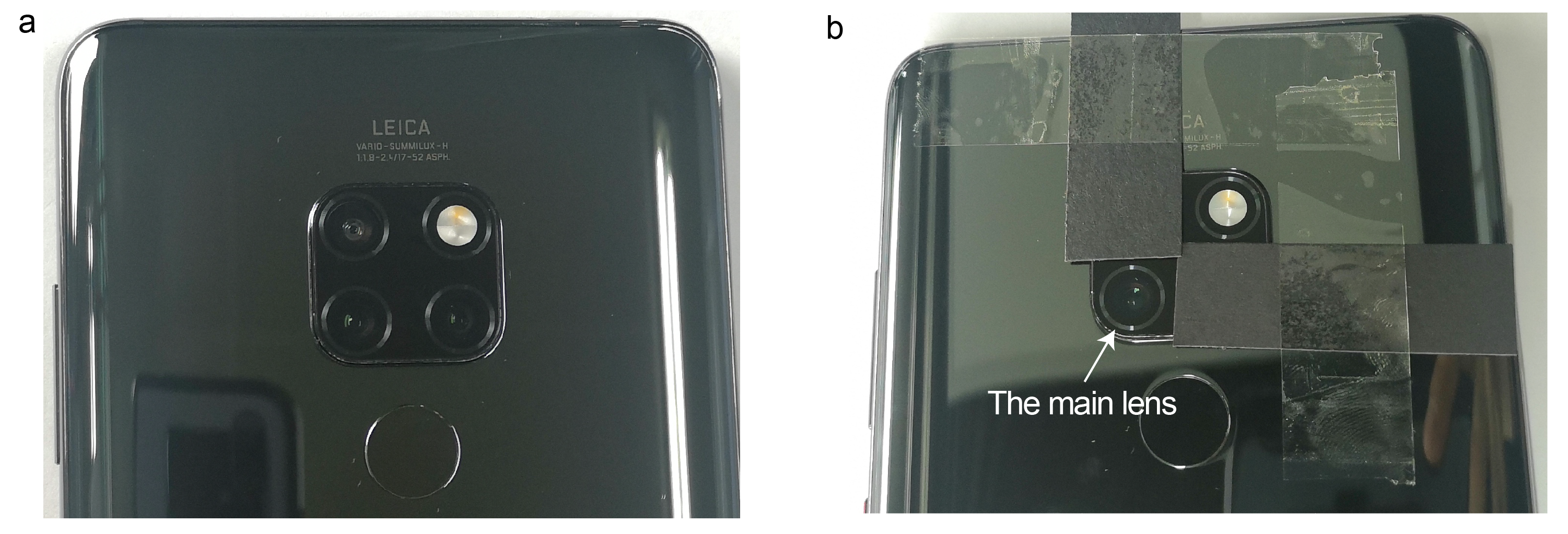


**Fig. S8. Camera phone used in the experiments. a,** The original multi-lens group**. b,** The single lens used in the experiments.


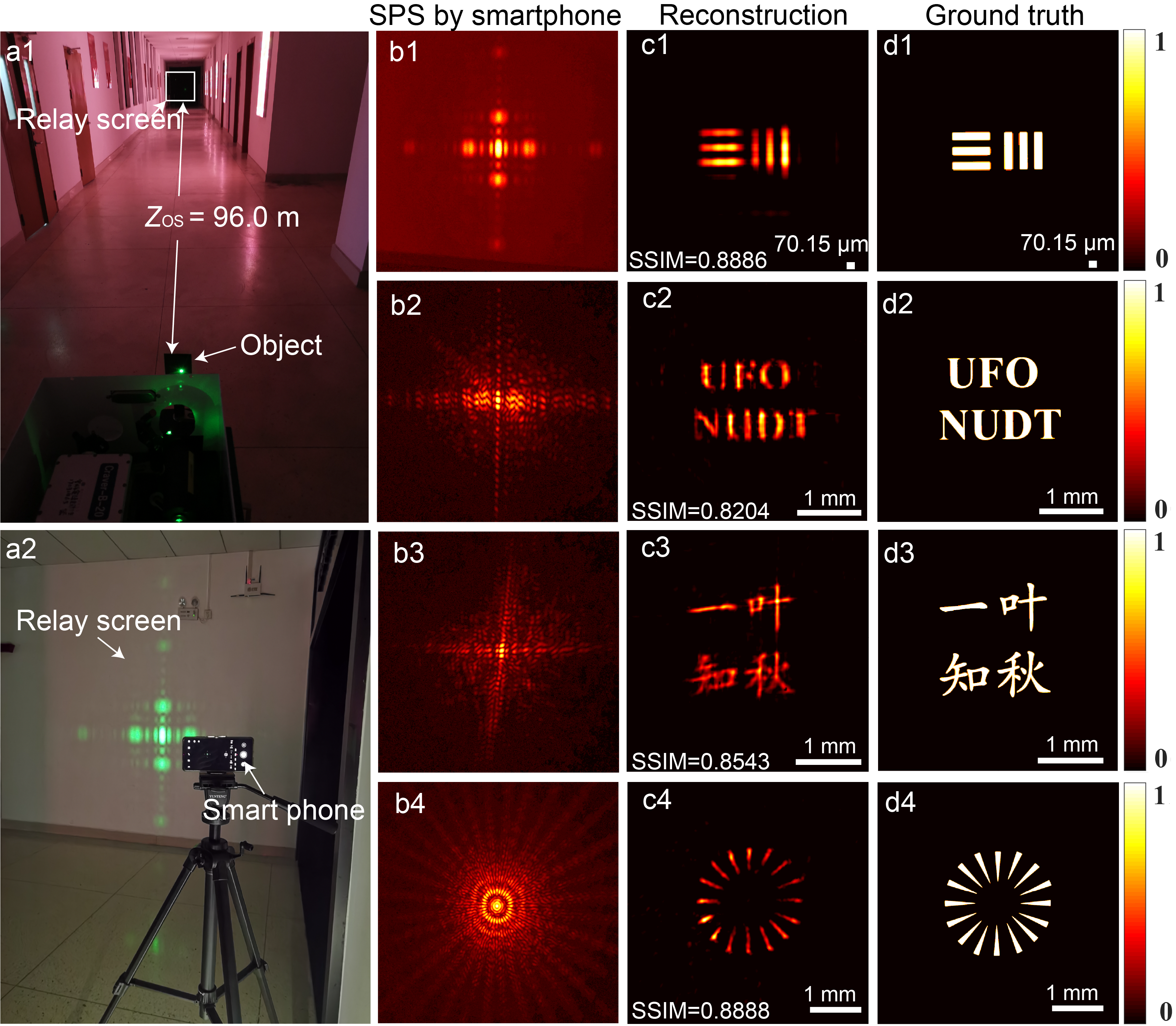


**Fig. S9. Long-distance, super-resolution rPMT with a smartphone (***Z*_OS_*=*96.0 m**). a1-a2**, Photograph of the experimental setup: a smartphone was used to capture the relay-projected SPS patterns. **b1-b4**, The normalized SPS patterns of the objects. **c1-c4**, Corresponding single-shot reconstructions from **b1-b4**. **d1-d4**, The corresponding ground truths.

**8. Random-matrix encoding masks used for Fig. 2f**

The random-matrix encoding mask with an element size of 9.26 μm used for Fig. 2 in the main text is shown in Fig. S10b. The corresponding reconstructions by our rPMT, i.e., Fig. 2f in the main text, are respectively shown in Fig. S10a. It can be observed that the matrix elements have been retrieved faithfully.


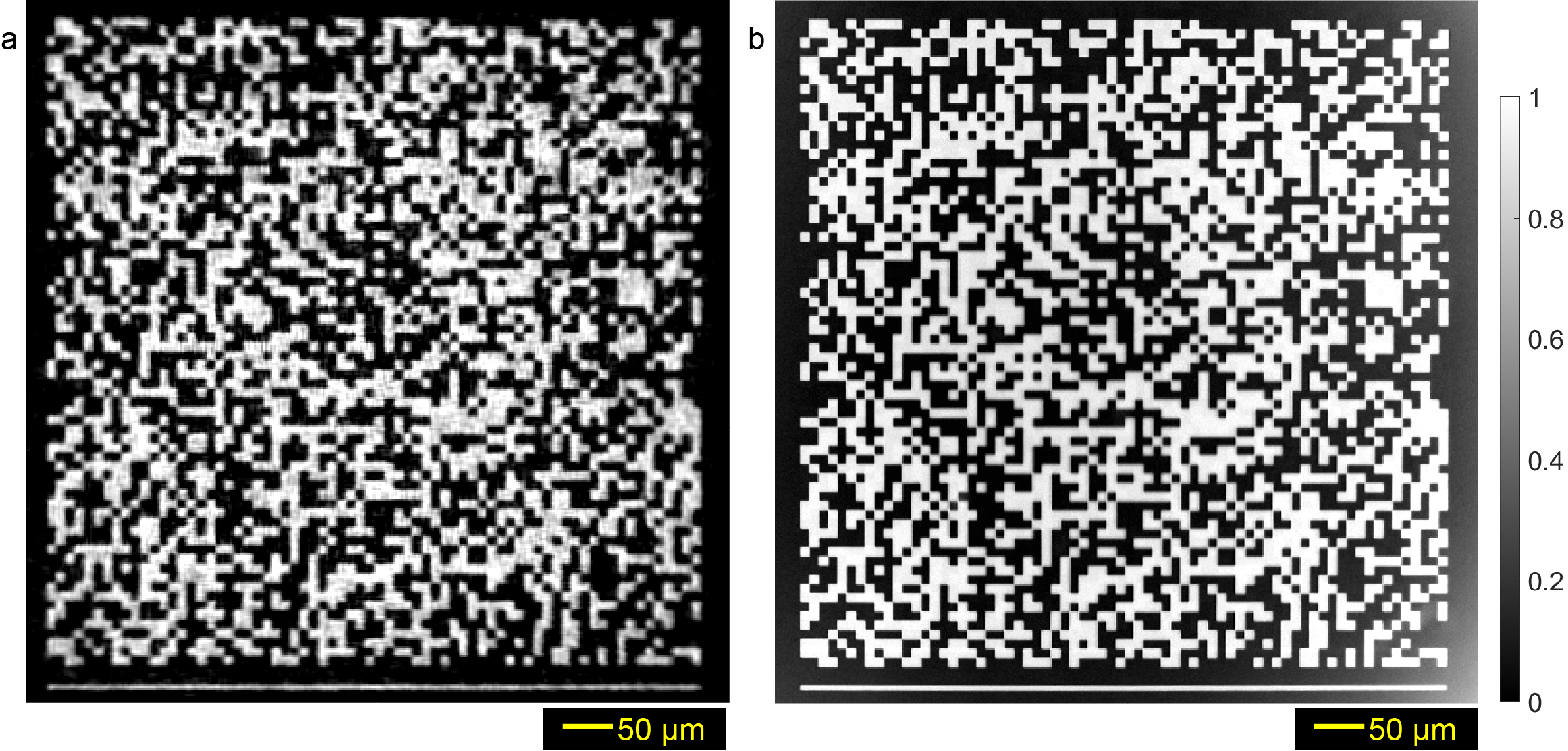


**Fig. S10. Random-matrix encoding mask** **with an element size of 9.26 μm for Fig. 2f in the main text. a,** the reconstructed encoding mask by our rPMT. **b,** the corresponding original object.

**9. Handmade reflective objects for long-distance rPMT**

The handmade reflective objects for long-distance rPMT (Fig. 4l, n in the main text) are shown in Fig. S11, which consist of groups of holes of various shapes made on mirrors.


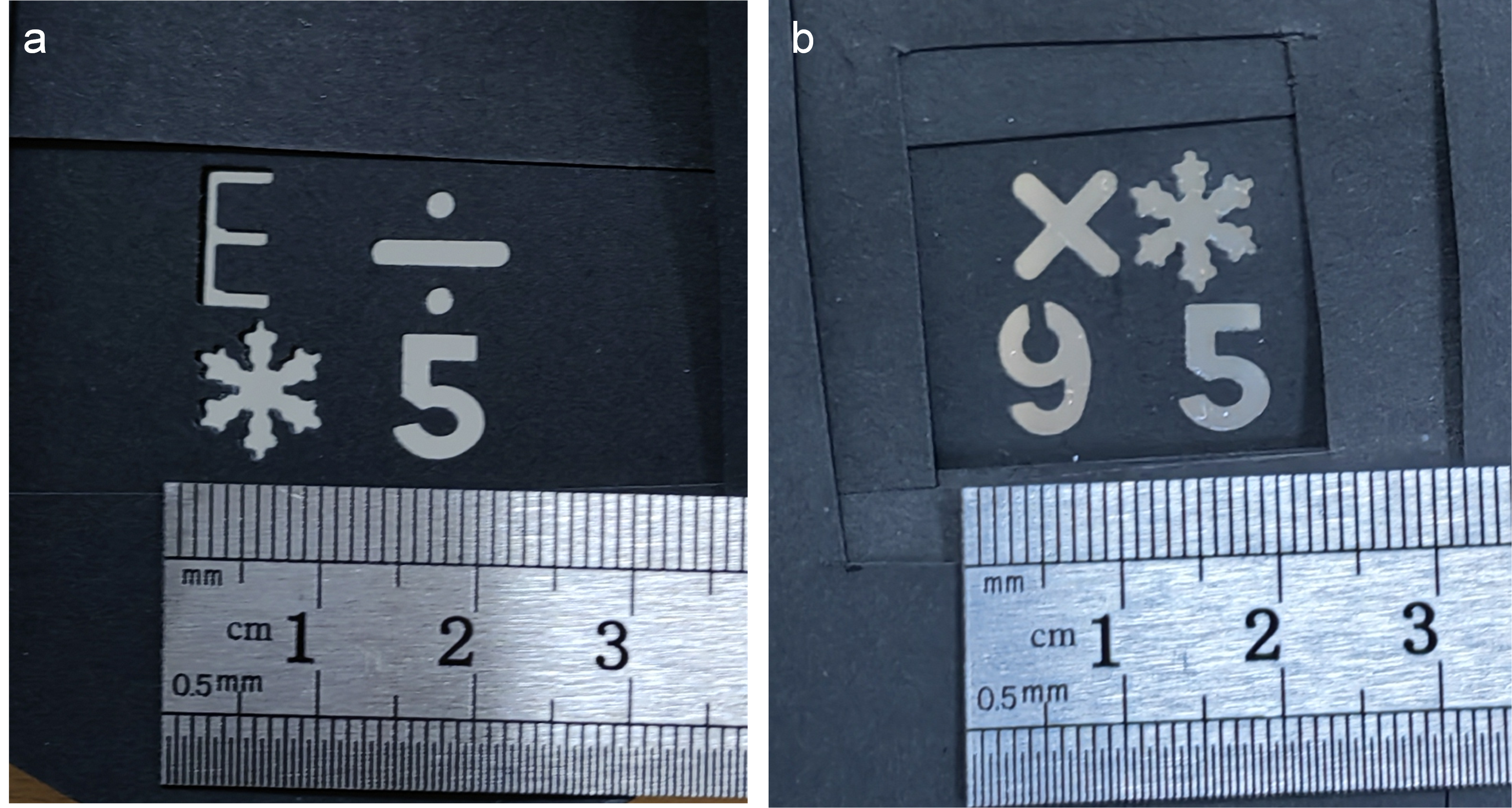


**Fig. S11 Two handmade reflective objects for long-distance rPMT in Fig. 4, l and n.**

**10. Homography-based distortion correction**

The non-line-of-sight configuration of the rPMT system results in a non-coaxial arrangement of the transmit-receive system, leading to distortion in the captured image of the SPS pattern. Specifically, in our rPMT experiments, the illuminating light was oriented orthogonally to the relay-projection screen, while the optical axis of the camera was not. Nevertheless, the angle between the camera's optical axis and the normal to the screen was relatively small, typically less than 80 mrad. Consequently, the distortion of the SPS patterns can be considered negligible. In instances where the angle between the camera's optical axis and the screen’s normal direction is sufficiently large to yield noticeable distortion in the SPS patterns, we propose a homography-based method for distortion correction as follows.

Images of a planar surface, such as a relay screen, can be associated from different viewpoints using a homography. This homography *H* is represented as a non-singular 3×3 matrix, which can be derived through standard camera calibration techniques^13, 14^.

$H=\left[\begin{matrix} h_{11} & h_{12} & h_{13} \\ h_{21} & h_{22} & h_{23} \\ h_{31} & h{}_{32} & h_{33} \end{matrix}\right]$ (S6)

The planar projective transformation from the target view to the reference view can be represented by

$\left[\begin{matrix} x' \\ y' \\ 1 \end{matrix}\right]=\left[\begin{matrix} h_{11} & h_{12} & h_{13} \\ h_{21} & h_{22} & h_{23} \\ h_{31} & h{}_{32} & h_{33} \end{matrix}\right]\left[\begin{matrix} x \\ y \\ 1 \end{matrix}\right]$ (S7)

or more briefly, $X'=HX$, where$X'=[x',y',1]^{T}$and$X=[x,y,1]^{T}$are the coordinates of an identical object point in the reference image and the target image, respectively.

The homography matrix *H* can be derived by mapping multiple reference points from a calibration target to the corresponding distorted image. In this study, we utilized a checkerboard affixed to the relay screen (see Fig. S12a) as the calibration target, with its distorted image presented in Fig. S12b. We subsequently extracted 48 reference points, indicated by red circles, from both the reference image (Fig. S12a) and the distorted image (Fig. S12b) to generate the homography matrix *H*. The corrected image, representing a frontal orthogonal view of Fig. S12b, is presented in Fig. S12c. Provided that the camera remains fixed in this position, the same homography matrix *H* can be employed to rectify the distortion of diffraction patterns captured by the rPMT system. However, if the camera is moved or rotated, a new calibration must be conducted.

In our rPMT experiment conducted at a projection distance of 26.4 m, we utilized an A3-sized checkerboard as the calibration target. The distorted and corrected images are illustrated in Fig. S12, d1 and d2, respectively. Additionally, the distorted and corrected SPS images are presented in Fig. S12, e1 and e2, with their corresponding reconstructed results shown in Fig. S12, f1 and f2. It is evident that the differences between the distorted and corrected images are minimal when the angle between the camera's optical axis and the normal direction of the screen is small. Given this observation, we did not perform homography-based distortion corrections on the experimental results presented in the main text; nevertheless, they still exhibit high Structural Similarity Index Measures (SSIMs), as shown in Fig. 4.


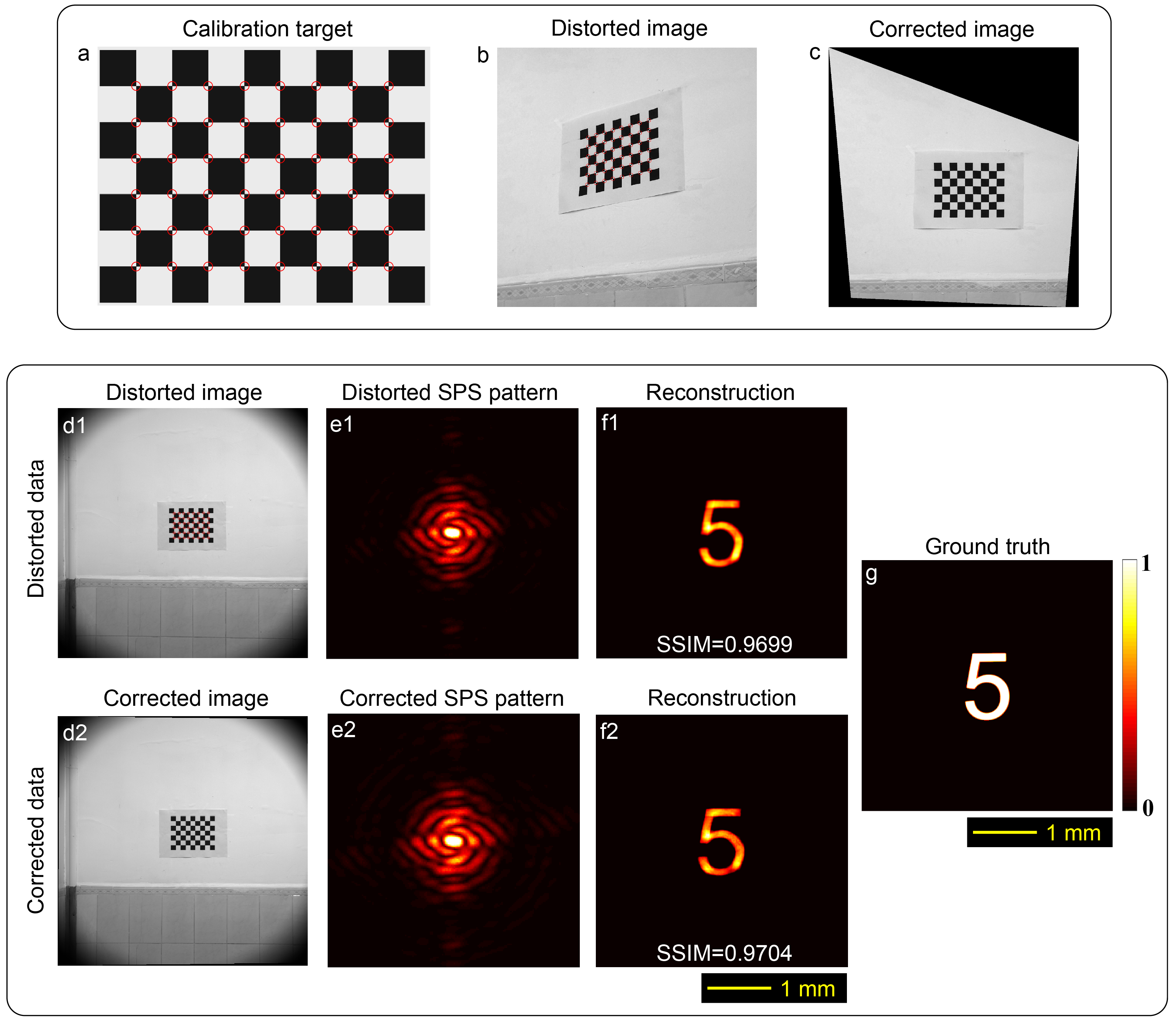


**Fig. S12. Homography-based distortion correction. a,** The calibration target**. b,** The distorted image of the calibration target. **c,** the corrected image, i.e., the frontal orthogonal view of **b**. **d1,** The distorted image of the relay screen with an A3-sized checkerboard. **d2,** the corrected image of **d1** using homograohy. **e1,** The distorted SPS pattern. **e2,** the corrected SPS pattern of **e1**.**f1** and **f2,** Reconstructed results from **e1** and **e2**, respectively. **g,** The ground truth.

**11. Runtimes under various conditions**

The Python implementations of the NCPP algorithm were executed on a GPU-equipped notebook computer (Micro-Star International Co., Ltd., Vector GP66 12UGS), which features an NVIDIA GeForce RTX 3070 Ti Laptop GPU with 5,888 cores and a 12th Generation Intel® Core™ i7 processor capable of reaching a maximum turbo frequency of 4.70 GHz. By averaging the reconstruction times of 200 frames, we determined that the average reconstruction time per frame can be reduced to 0.827 seconds through parallel GPU computing. The runtimes under various conditions are presented in Supplementary Table S2.

**Table S2. Runtimes under various conditions**

|  | GPU computing | | CPU computing | |
| --- | --- | --- | --- | --- |
| Parallel computation | 1 | 5 | 1 | 5 |
| Total reconstruction time of 200 frames (second) | 211.105 | 165.312 | 4430.804 | 1488.015 |
| Average reconstruction time of one frame (second) | 1.056 | **0.827** | 22.154 | 7.44 |

**12. Robustness tests of rPMT**

Our method demonstrates strong robustness concerning the object-to-screen distance *Z*_OS_. We first evaluate its robustness through long-distance rPMT experiments. As illustrated in Fig. S13, the precise value of *Z*_OS_ is 96.0 m, resulting in a clear reconstructed image, as shown in Fig. S13g. Generally, the quality of the reconstruction deteriorates with increasing deviations from the precise *Z*_OS_. However, it is noteworthy that a deviation of *Z*_OS_ of 2 m or less still maintains clarity. In contrast, deviations exceeding 4 m lead to an increase in artifacts and noise. This indicates that long-distance rPMT exhibits significant robustness with respect to *Z*_OS_. The reconstructible range of the *Z*_OS_ value, which allows for the production of a reconstructed object instead of resulting in complete reconstruction failure, exceeds 11 m/96 m=11.5% of the precise *Z*_OS_ value, suggesting a considerable degree of robustness. This robustness plays a critical role in determining the search efficiency of *Z*_OS_ when the precise value of *Z*_OS_ is unknown or difficult to be measured accurately. Similarly, as shown in Fig. S14, the same conclusion applies to meter-scale rPMT-based microscopic imaging.

It is important to note that, although we can reconstruct the object image within a relatively large deviation, a larger deviation in *Z*_OS_ will lead to a greater discrepancy in the estimated size of the object. As shown in Fig. S13a-l, as the input value of *Z*_OS_ in the NCPP algorithm increases, the sizes of the reconstructed objects also increase. However, among these results, only the precise value of *Z*_OS_ yields an accurate estimation of the object size. This phenomenon can be explained by the relationship between the object plane and the observation plane (i.e., the relay screen plane), expressed as $M\Delta x_{0}=\frac{\lambda Z_{\text{OS}}}{\Delta x}$ (see Eq. S3 in the Supplementary materials), where $\Delta x_{0}$ and $\Delta x$ represent the sampling intervals of the object plane and observation plane, respectively, and *M* denotes the row sampling number. The term $M\Delta x_{0}$ signifies the size of the object plane. Assuming other sources of error are negligible, the size deviation of the object is directly proportional to the deviation in the object-to-screen distance *Z*_OS_, i.e., $\delta\left(M\Delta x_{0}\right)=\frac{\lambda\delta\left(Z_{\text{OS}}\right)}{\Delta x}$. In other words, the object size and the object-to-screen distance *Z*_OS_ exhibit the same relative error. Furthermore, we can conclude from this analysis that when *Z*_OS_ is unknown, prior information about the object size can assist in estimating the object-to-screen distance *Z*_OS_. For instance, as shown in Fig. S13, a range of object sizes can correspondingly yield a range of *Z*_OS_ values.


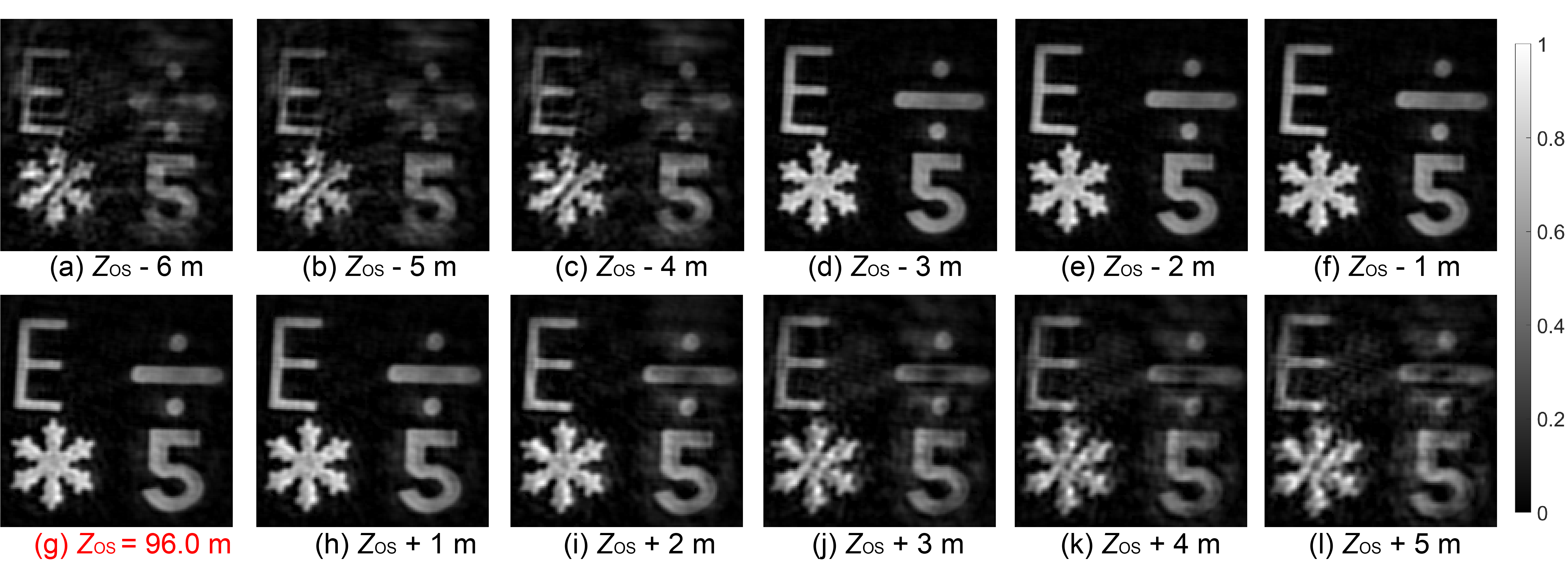


**Fig. S13. Robustness test of the object-to-screen distance *Z*os.** In this rPMT experiment (Fig. 4m in the main text), the precise value of *Z*os is 96.0 m. **a-l** present the corresponding reconstructed results for varying values of *Z*os.


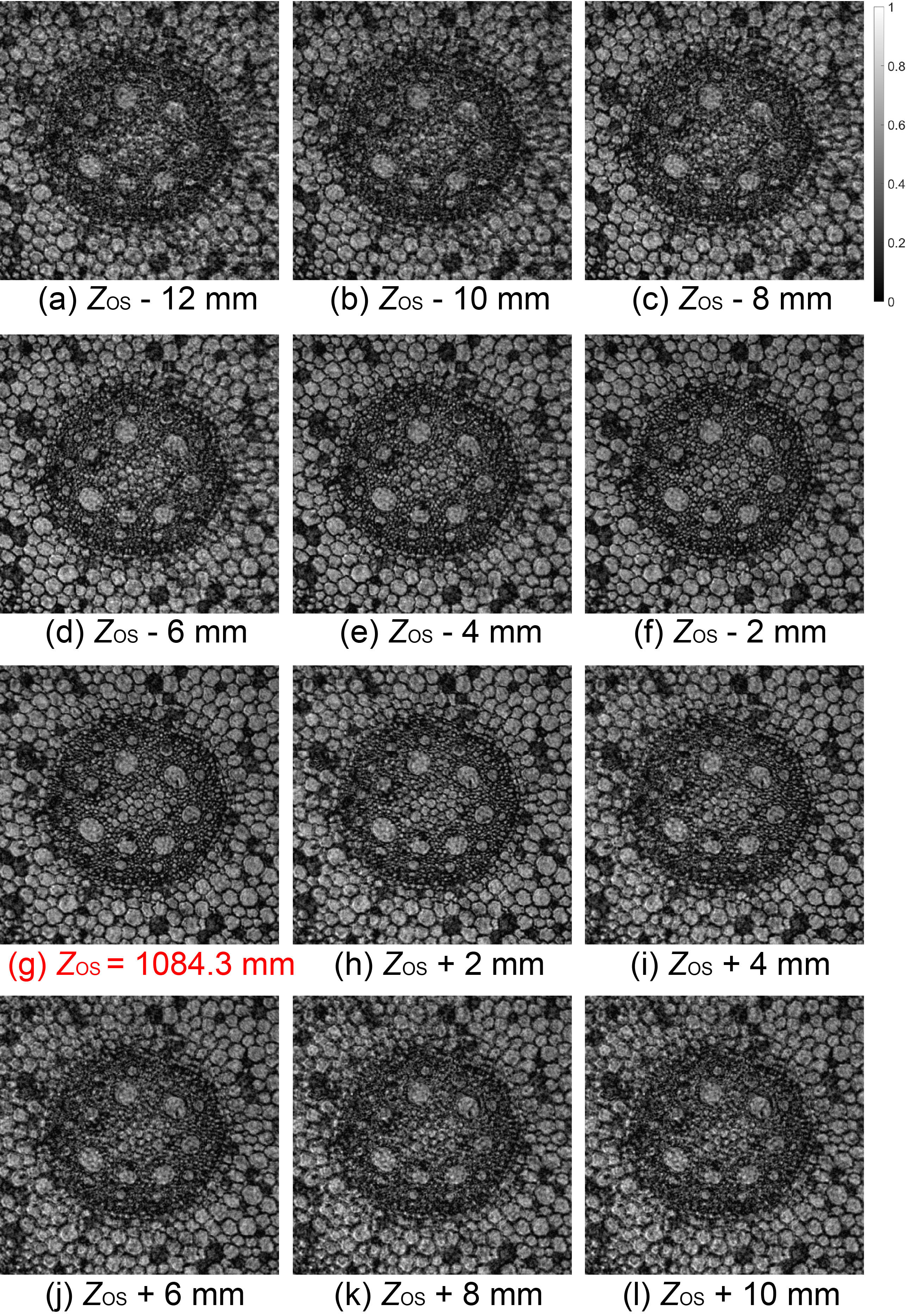


**Fig.S14. Robustness test of the object-to-screen distance *Z*os.** In this rPMT experiment (Fig. 3c in the main text), the precise value of *Z*os is 1084.3 mm. **a-l** present the corresponding reconstructed results for varying values of *Z*os.

**13. Signal to noise ratio analysis**

In the following section, we will analyze the SNR of the SPS image obtained through a rPMT system, along with strategies to enhance this ratio. Additionally, we use a set of USAF-type line pairs as an example to outline the constraints necessary for successful reconstruction, including the requirements for relay screen size and the SNR constraints of the system.

**① Diffracted light power received by a detector pixel**

In a rPMT system, the diffracted light power received by a detector pixel can be calculated as follows:

$P_{\text{R,rPMT}}=I_{Z}(x,y)\Delta x^{2}\cdot\rho_{\text{dr}}\cdot\frac{\pi(L_{\text{C}}/2)^{2}}{2\pi Z_{\text{CS}}^{2}}$ (S8)

where $(x,y)$ is the observation-plane (relay screen) coordinates, $I_{Z}(x,y)$is the intensity distribution of the object’s diffracted light in the observation plane, $\rho_{\text{dr}}$ denotes the diffuse reflectance of the relay screen surface (approximated as a Lambertian reflector), $L_{C}$ represents the aperture diameter of the camera lens, $Z_{\text{CS}}$ is the camera-to-screen distance and $\Delta x$ represents the sampling interval on the relay screen. The sampling interval on the relay screen $\Delta x$ and the pixel size of the detector $\Delta x_{\text{pix}}$ can be approximately linked by $\Delta x=Z_{\text{CS}}\Delta x_{\text{pix}}/f$, in which *f* is the focal length of the lens.

The intensity distribution of the diffracted light on the relay screen $I_{Z}(x,y)$ can be expressed as

$I_{Z}(x,y)=\frac{I_{0}\rho_{\text{obj}}I_{\text{relat}}(x,y)}{Z_{\text{OS}}^{2}}$ (S9)

where $I_{0}$ is the intensity of coherent illuminating light received by the object，*Z*_OS_ is the object-to-screen distance, $\rho_{\text{obj}}$is the transmittance or reflectance of the object. $I_{\text{relat}}(x,y)/Z_{\text{OS}}^{2}$ represents the relative intensity distribution of the diffracted light on the relay screen, which is a dimensionless quantity. Here, $1/Z_{\text{OS}}^{2}$ is isolated from the relative intensity distribution to facilitate the analysis of the effects of *Z*_OS_.

Therefore, the power received by a detector pixel can be simplified as

$P_{\text{R,rPMT}}=\frac{I_{0}\rho_{\text{obj}}\rho_{\text{dr}}\Delta x_{\text{pix}}^{2}L_{C}^{2}}{8f^{2}Z_{\text{OS}}^{2}}I_{\text{relat}}(x,y)$ (S10)

It should be pointed that $I_{\text{relat}}(x,y)$ relies on factors such as the shape and area of the aperture object and it cannot typically be expressed analytically, except for a few standard geometric figures.

**② Gray level of diffracted light** **signal in a detector pixel**

The expected gray level of diffracted light signal received by a detector pixel in the rPMT system can further be estimated as follows:

$\begin{matrix} \bar{g}_{\text{rPMT}}=\frac{P_{\text{R,rPMT}}T_{\text{ex}}\xi_{\text{lens}}Q_{\text{e}}}{\varepsilon Q_{\text{a}}} \\ =\frac{T_{\text{ex}}\xi_{\text{lens}}Q_{\text{e}}}{\varepsilon Q_{\text{a}}}\cdot\frac{I_{0}\rho_{\text{obj}}\rho_{\text{dr}}\Delta x_{\text{pix}}^{2}L_{\text{C}}^{2}}{8f^{2}Z_{\text{OS}}^{2}}I_{\text{relat}}(x,y) \end{matrix}$ (S11)

where $T_{\text{ex}}$ represents the exposure time of the detector, $\xi_{\text{lens}}$ represents the optical efficiency of the lens ($0\leq\xi_{\text{lens}}\leq1$), $Q_{e}$ represents the quantum efficiency of the detector ($0\leq Q_{\text{e}}\leq1$), $Q_{\text{a}}$ represents the quantization accuracy (electrons per count) of the detector, $\varepsilon=hc/\lambda$ (*h* is the Planck constant and c is the speed of light) is the single photon energy.

Eq. S11 indicates the method for enhancing the SNR of diffracted light signal. Specifically, this enhancement can be achieved by increasing the laser power and exposure time $T_{\text{ex}}$, extending the lens aperture $L_{\text{C}}$, enlarging the transmittance or reflectance $\rho_{\text{obj}}$ of the object, increasing the diffuse reflectance $\rho_{\text{dr}}$ of the relay screen surface, enlarging the detector pixel size $\Delta x_{\text{pix}}$, reducing the object-to-screen distance *Z*_OS_ and so on.

**③ Constraints required for line pair reconstruction**

$I_{\text{relat}}(x,y)$ relies on factors such as the shape and area of the object. Herein, taking a set of line pairs (rectangular apertures with a line width of *a*, a length of *b*, a center-to-center distance of *d* and a total of *N* lines) as an example, under far-field diffraction, $I_{\text{relat}}(x,y)$ can be analytically expressed as:

$I_{\text{relat}}(x,y)=\frac{a^{2}b^{2}}{\lambda^{2}}\cdot\left(\frac{\sin\frac{\pi ax}{\lambda Z_{\text{OS}}}}{\frac{\pi ax}{\lambda Z_{\text{OS}}}}\right)^{2}\left(\frac{\sin\frac{N\pi dx}{\lambda Z_{\text{OS}}}}{\sin\frac{\pi dx}{\lambda Z_{\text{OS}}}}\right)^{2}\cdot\left(\frac{\sin\frac{\pi by}{\lambda Z_{\text{OS}}}}{\frac{\pi by}{\lambda Z_{\text{OS}}}}\right)^{2}$ (S12)

For a typical USAF-type line pair, the parameters are defined as $d=2a$ and $N=3$. To successfully reconstruct the line pair target from its SPS image, it is necessary to capture the first-order bright fringe located at $x_{1}=\pm\lambda Z_{\text{OS}}/d$ as well as the zeroth-order bright fringe in the diffraction pattern. Therefore, the size of the relay screen $L_{\text{s}}$ must be sufficiently large to encompass the first-order bright fringe:

$L_{\text{s}}\geq2x_{1}=2\lambda Z_{\text{OS}}/d$ (S13)

In fact, this minimum threshold of the relay screen size $L_{\text{s,min}}=2\lambda Z_{\text{OS}}/d$ corresponds to the Abbe diffraction limit of $\delta=2\lambda Z_{\text{OS}}/L_{\text{s,min}}=d$, which is exactly equal to the center-to-center distance of the line pair.

To achieve successful target reconstruction, in addition to the above requirements regarding the size of the relay screen, there are also specific demands on the imaging SNR of the first-order bright fringe. The relative intensity of the first-order bright fringe can be calculated by:

$I_{\text{relat,1}}(x=x_{1},y=0)=\frac{36a^{2}b^{2}}{\pi^{2}\lambda^{2}}=\frac{4}{\pi^{2}}I_{\text{relat,0}}(x=0,y=0)$ (S14)

Therefore, according to Eq.S11, the expected gray level of the first-order bright fringe can be expressed as:

$\bar{g}_{\text{rPMT,1}}=\frac{T_{\text{ex}}\xi_{\text{lens}}Q_{\text{e}}}{\varepsilon Q_{\text{a}}}\cdot\frac{I_{0}\rho_{\text{obj}}\rho_{\text{dr}}\Delta x_{\text{pix}}^{2}L_{\text{C}}^{2}}{8f^{2}Z_{\text{OS}}^{2}}\cdot\frac{36a^{2}b^{2}}{\pi^{2}\lambda^{2}}$ (S15)

Consequently, the SNR of the first-order bright fringe can be expressed as:

$SNR=\frac{\bar{g}_{\text{rPMT,1}}}{\sigma_{\text{noise}}}$ (S16)

where $\sigma_{\text{noise}}=\sqrt{\sigma_{\text{shot}}^{2}+\sigma_{\text{dark}}^{2}+\sigma_{\text{read}}^{2}+\sigma_{\text{bg}}^{2}}$represents the noise standard deviation. Here, $\sigma_{\text{shot}}$,$\sigma_{\text{dark}}$,$\sigma_{\text{read}}$and$\sigma_{\text{bg}}$ represents the standard deviations of photon shot noise, dark current noise, readout noise, and background (ambient light) noise, respectively.

The SNR must exceed a specific threshold to achieve target reconstruction. Our tests indicate that this threshold is approximately 10; thus, when the SNR of the high-frequency components of SPS image exceeds 10, information extraction and target reconstruction can be successfully accomplished.

In conclusion, to achieve successful target reconstruction of such a set of line pairs with a center-to-center distance of *d*=2*a*, the following two inequality constraints must be satisfied.

$\left\{\begin{matrix} L_{\text{s}}\geq2\lambda Z_{\text{OS}}/d \\ SNR=\frac{\bar{g}_{\text{rPMT,1}}}{\sigma_{\text{noise}}}\geq10 \end{matrix}\right.$ (S17)

where $\bar{g}_{\text{rPMT,1}}$ can be represented by parameters related to the laser, the target, the detector, and the distances as Eq. S15.

**References**

1. Goodman, J. Introduction to Fourier Optics. 3rd ed. Roberts and Company Publishers: Englewood, 2004.

2. Fienup, J. R. Phase retrieval algorithms: a comparison. *Appl. Optics*. **21**, 2758-2769 (1982).

3. Fienup, J. R. Reconstruction of an object from the modulus of its Fourier transform. *Opt. Lett.* **3**, 27-29 (1978).

4. Hofer, M. et al. Wide field fluorescence epi-microscopy behind a scattering medium enabled by speckle correlations. *Opt. Express*. **26**, 9866-9881 (2018).

5. Zhang, F. C. & Rodenburg, J. M. Phase retrieval based on wave-front relay and modulation. *Phys. Rev. B*. **82**, 121104 (2010).

6. Francis T. S. et al. Introduction to information optics. Academic Press: San Diego, 2001.

7. Fienup, J. R., Crimmins, T. R. & Holsztynski, W. Reconstruction of the support of an object from the support of its autocorrelation. *JOSA*. **72**, 610-624 (1982).

8. Tang, W. S. et al. Single-shot coherent power-spectrum imaging of objects hidden by opaque scattering media. *Appl. Optics*. **58**, 1033-1039 (2019).

9. Feng, S. C. et al. Correlations and fluctuations of coherent wave transmission through disordered media. *Phys. Rev. Lett.* **61**, 834-837 (1988).

10. Freund, I., Rosenbluh, M. & Feng, S. C. Memory effects in propagation of optical waves through disordered media. *Phys. Rev. Lett.* **61**, 2328-2331 (1988).

11. Judkewitz, B. et al. Translation correlations in anisotropically scattering media. *Nat. Phys.* **11**, 684-689 (2015).

12. Katz, O. et al. Non-invasive single-shot imaging through scattering layers and around corners via speckle correlations. *Nat. Photonics*. **8**, 784-790 (2014).

13. Zhang, Z. Y. Flexible camera calibration by viewing a plane from unknown orientations. in *Proceedings of the Seventh IEEE International Conference on Computer Vision.* Kerkyra, Greece: IEEE, 1999, 666–673.

14. Hartley, R. et al. Multiple View Geometry in Computer Vision. Cambridge University Press: New York, 2003.
